# Supplementary material for: A novel mouse model of mitochondrial disease exhibits juvenile-onset severe neurological impairment due to parvalbumin cell mitochondrial dysfunction
Source: Commun Biol. 2023 Oct 23;6:1078. doi: 10.1038/s42003-023-05238-7 (PMC10593770; doi:10.1038/s42003-023-05238-7)
Supplement: Supplementary file 2 — Supplemental Material [file 42003_2023_5238_MOESM2_ESM.pdf]

Supplementary Information

Supplementary Table 1. Clinical scoring criteria and scale designed to monitor symptom development and progression in rodents.

Feature	Description (score)					
Coat appearance	Normal (0)	Piloerection (1)	Porphyrin (2)			
Breathing	Normal (0)	Rapid (1)	Laboured (2)	Cyanosis (3)		
Posture	Normal (0)	Twitching (1)	Some huddling and twitching (2)	Huddling and twitching (3)		
Weight	If weight loss exceeds 10%, soaked diet is provided, if weight loss is 20% or greater, mice are to be humanely euthanized.					
Wobble	None (0)	Wobbles while stationery (1)	Both wobbles and falls (2)	Falls from side to side (3)	Loss of righting reflex (4)	
Modified Racine Scale	No response (0)	Freezing (1)	Head nodding, isolated twitches (2)	Orofacial seizure (3)	Clonic seizure (4)	Tonic seizure (5)

Supplementary Table 2. Quantitative real-time PCR master mix per reaction.

Reagent	Volume per 20 µl reaction (µl)
dH_2O	7.4
MT-ND5 forward primer (10 µM)	0.6
MT-ND5 reverse primer (10 µM)	0.6
iTaq	10
MT-ND5 probe (10 µM)	0.4

Supplementary Table 3. List of primary and secondary antibodies used for FFPE immunohistochemistry with Vector Elite ABC kit and DAB chromogen.

**Antibody**	**Optimal dilution**	**Company code**	**Isotype**	**Secondary antibody used**
Iba-1	1:2,000	Wako 019-19741	Rabbit IgG	Biotinylated anti-rabbit
GFAP	1:15,000	Dako Z0334	Rabbit IgG	Biotinylated anti-rabbit
Parvalbumin (PV)	1:6,000	Swant PV27	Rabbit IgG	Biotinylated anti-rabbit
Calbindin (CB)	1:6,000	Swant 300	Rabbit IgG	Biotinylated anti-rabbit
GAD65-67	1:12,000	Sigma Aldrich G5163	Rabbit IgG	Biotinylated anti-rabbit
c-Fos	1:2,000	Abcam ab222699	Rabbit IgG	Biotinylated anti-rabbit

Supplementary Table 4. List of primary antibodies used for immunofluorescence.

**Mitochondrial markers** (Antigen retrieval: EDTA pH 8.0)			
**Antibody**	**Optimal dilution**	**Company code**	**Isotype**
NDUFB8	1:100	Abcam ab110242	Mouse IgG1
NDUFA13	1:100	Abcam ab110240	Mouse IgG2b
SDHA	1:200	Abcam ab14715	Mouse IgG1
UqCRC2	1:100	Abcam ab14745	Mouse IgG1
COXI	1:200	Abcam ab14705	Mouse IgG2a
COXIV	1:200	Abcam ab14744	Mouse IgG2a
ATP-B	1:200	Abcam ab14730	Mouse IgG1
Porin	1:200	Abcam ab14734	Mouse IgG2b
Pyruvate carboxylase (EDTA pH 9.0)	1:100	Sigma-Aldrich HPA043922	Rabbit IgG
**CNS markers and other proteins** (Antigen retrieval: sodium citrate pH 6.0)			
**Antibody**	**Optimal dilution**	**Company code**	**Isotype**
PV	1:500 (biotinylated) or 1:100	Swant PV27	Rabbit IgG
PV (either retrieval)	1:100	Sigma-Aldrich P3088	Mouse IgG1
Tyrosine hydroxylase	1:100	Sigma-Aldrich T8700	Rabbit IgG
PGC1 (EDTA pH 8.0)	1:100	Sigma-Aldrich AB3242	Rabbit IgG
GAD65-67 (EDTA pH 8.0)	1:100	Sigma –Aldrich G5163	Rabbit IgG

Supplementary Table 5. List of AlexaFluor-conjugated secondary antibodies used for immunofluorescence at 1:100 dilution.

Antibody	AlexaFluor excitation wavelength (nm)
Goat streptavidin	405
Goat anti-rabbit	350 or 405 or 488 or 546
Goat anti-mouse IgG1	488 or 546 or 647
Goat anti-mouse IgG2a	488 or 546 or 594
Goat anti-mouse IgG2b	546 or 647

ID	Source	Sex	Age	Fixation	PMI delay	Cause of death
C1	Edinburgh SD008/17	F	71	20d	96	1a Ischaemic and hypertensive heart disease; 1b diabetes mellitus
C2	Edinburgh SD010/17	M	57	4d	64	1a Ischaemic and hypertensive heart disease
C3	Edinburgh SD012/17	F	71	4d	95	1a Plastic bag suffocation
C4	Edinburgh SD013/17	M	51	16d	52	1a Coronary artery atheroma
C5	Edinburgh SD043/16	M	47	10d	67	1a Ischaemic heart disease with cardiac enlargement and coronary artery thrombus
C6	Edinburgh SD048/16	M	49	13d	94	1a Ischaemic heart disease, 1b Coronary artery atherosclerosis
C7	Edinburgh SD051/16	M	57	10d	110	1a Coronary artery atheroma and thrombosis
C8	NBTR	M	55	7w	50	Carcinomatosis, cancer oesophagus
C9	NBTR	F	65	9w	47	Metastatic ovarian cancer; Non-Hodgkins lymphoma
C10	NBTR	F	59	9w	34	1a Multi organ failure, 1b ischaemic leg, 1c peripheral vascular disease, 2a PE stroke, 2b MI, 2c mesenteric ischaemia and short bowel syndrome
C11	NBTR	M	59	9w	78	Metastatic sigmoid cancer
C12	NBTR	M	66	9w	56	Exacerbation of COPD
C13	NBTR	M	66	10w	47	Metastatic rectal cancer (metastasis in liver and lung)
C14	NBTR	F	18	8w	81	Malignant hyperthermia secondary to MDMA
C15	NBTR	M	47	6w	29	1a. Malignant hypercalcemia, 1b. Metastatic penile cancer, 2. Pulmonary embolism
C16	NBTR	M	56	15w	100	Cardiomyopathy

Supplementary Table 6. Demographic description of neurologically intact control cohort included in *post-mortem* primary visual cortex (BA17) study. COPD – chronic obstructive pulmonary disease; MI – myocardial infarction; PE – pulmonary embolism.

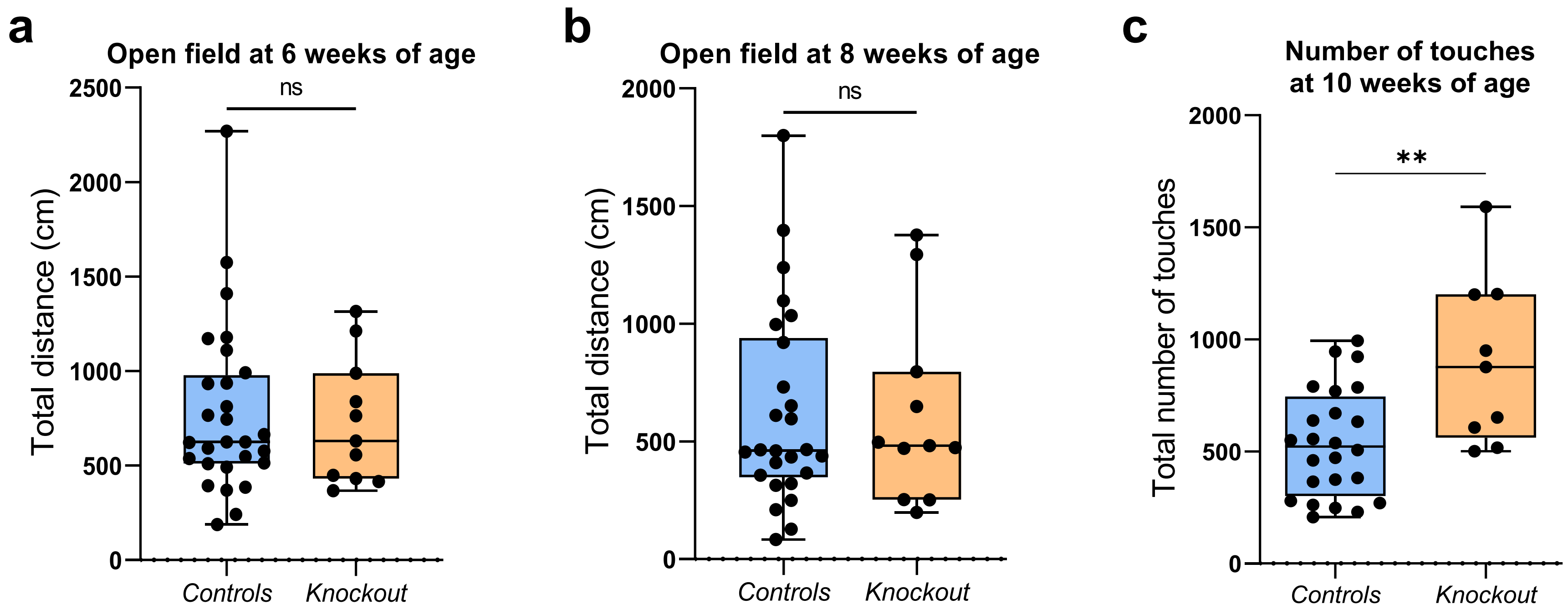

Supplementary Fig. 1: No changes in open field test at 6 and 8 weeks of age in the knockout mice in relation to littermate controls, however the number of foot touches of knockout mice is increased at 10 weeks of age, corroborating increased distance travelled at this age. At **a** 6 and **b** 8 weeks of age, no statistically significant difference was observed between the two groups (P = 0.8883 and P = 0.7272, respectively, Mann-Whitney test). **c** Total number of foot touches within the 5 minutes of open field test by homozygous mutant (n = 9) and control littermates (n = 24) at 10 weeks of age, consistent with an increased distance travelled data at 10 weeks of age (Fig. 1d). This displays further evidence that homozygous mutant animals exhibit hyperlocomotion compared to their littermates (P = 0.0023, Student's t-test).

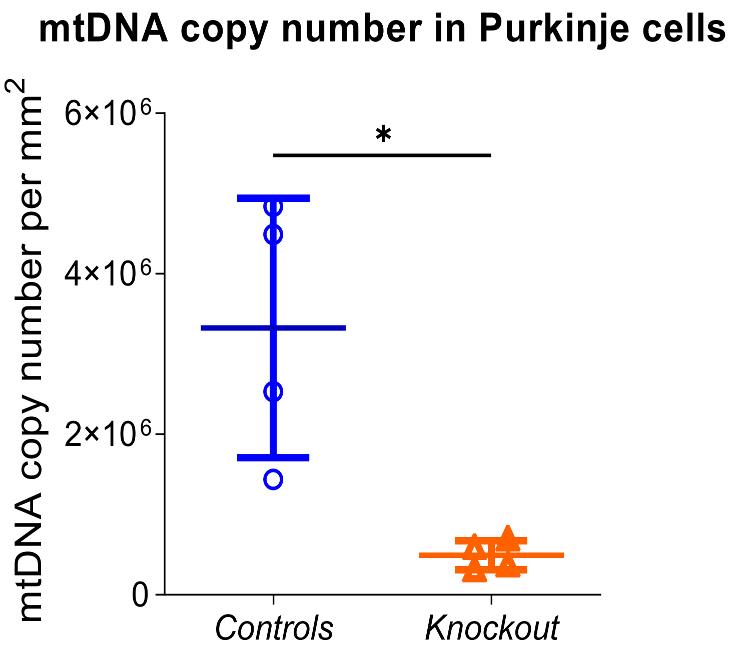

Supplementary Fig. 2: mtDNA depletion in pooled Purkinje neurons per mouse. Mean mitochondrial DNA copy number was significantly lower in Purkinje neurons in the knockout group in comparison to littermate control animals (P = 0.0132, Student's t-test; n = 4 mice per group). Each point indicates pooled mtDNA copy number per mouse per mm^2 of laser capture microdissection area that was occupied by 50 Purkinje neurons. Graph indicates mean ± SD.

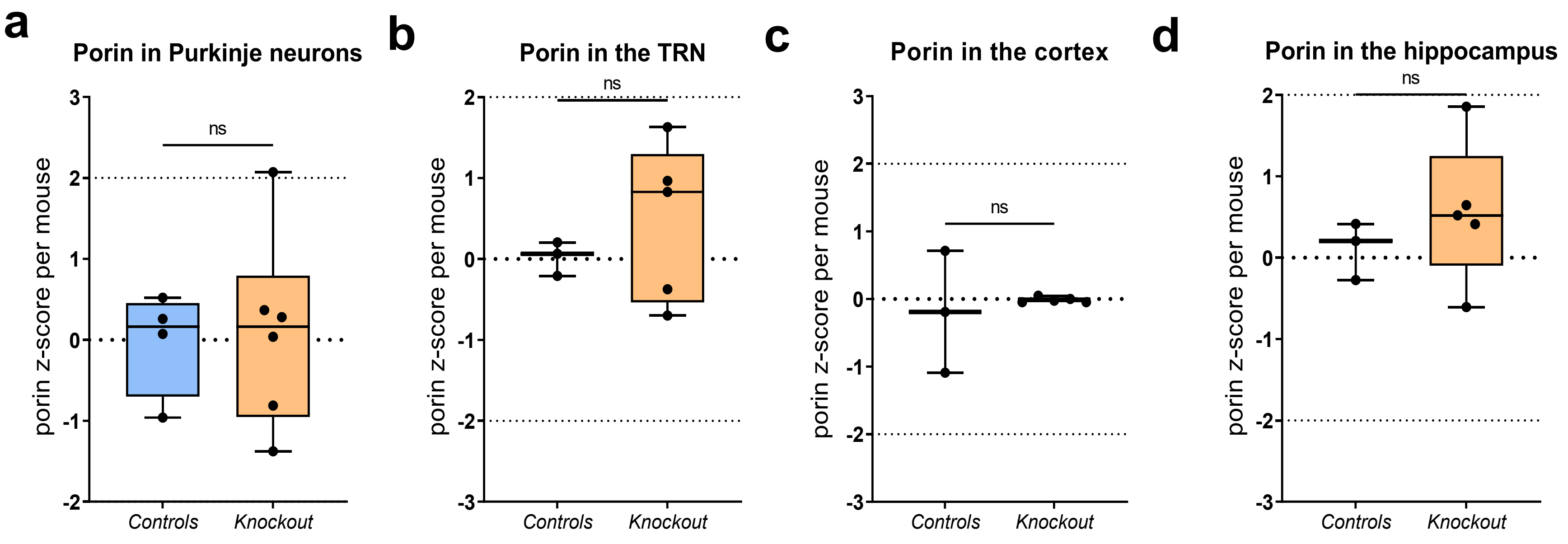

Supplementary Fig. 3: Brain region-dependent changes in mitochondrial mass in PV$^+$ neurons of *PV^{cre}Tfam$^{-/-}$* mice. a No difference in mean porin z-scores between groups in Purkinje neurons (*P* = 0.8569, *t*-test; *n* = 97 neurons from 4 control and *n* = 82 neurons from 3 knockout mice). **b** Mean porin z-score was not significantly greater in the PV$^+$ cells of the TRN region in the knockout mice (*P* = 0.4726, *t*-test; *n* = 149 neurons from 4 control and *n* = 76 neurons from 3 knockout mice), **c** PV$^+$ interneurons of cortical regions (*P* = 0.6632, *t*-test; *n* = 35 neurons from 2 control and *n* = 37 neurons from 2 knockout mice), and **d** hippocampus (*P* = 0.4391, *t*-test; *n* = 18 neurons from 4 control and *n* = 25 neurons from 3 knockout mice).

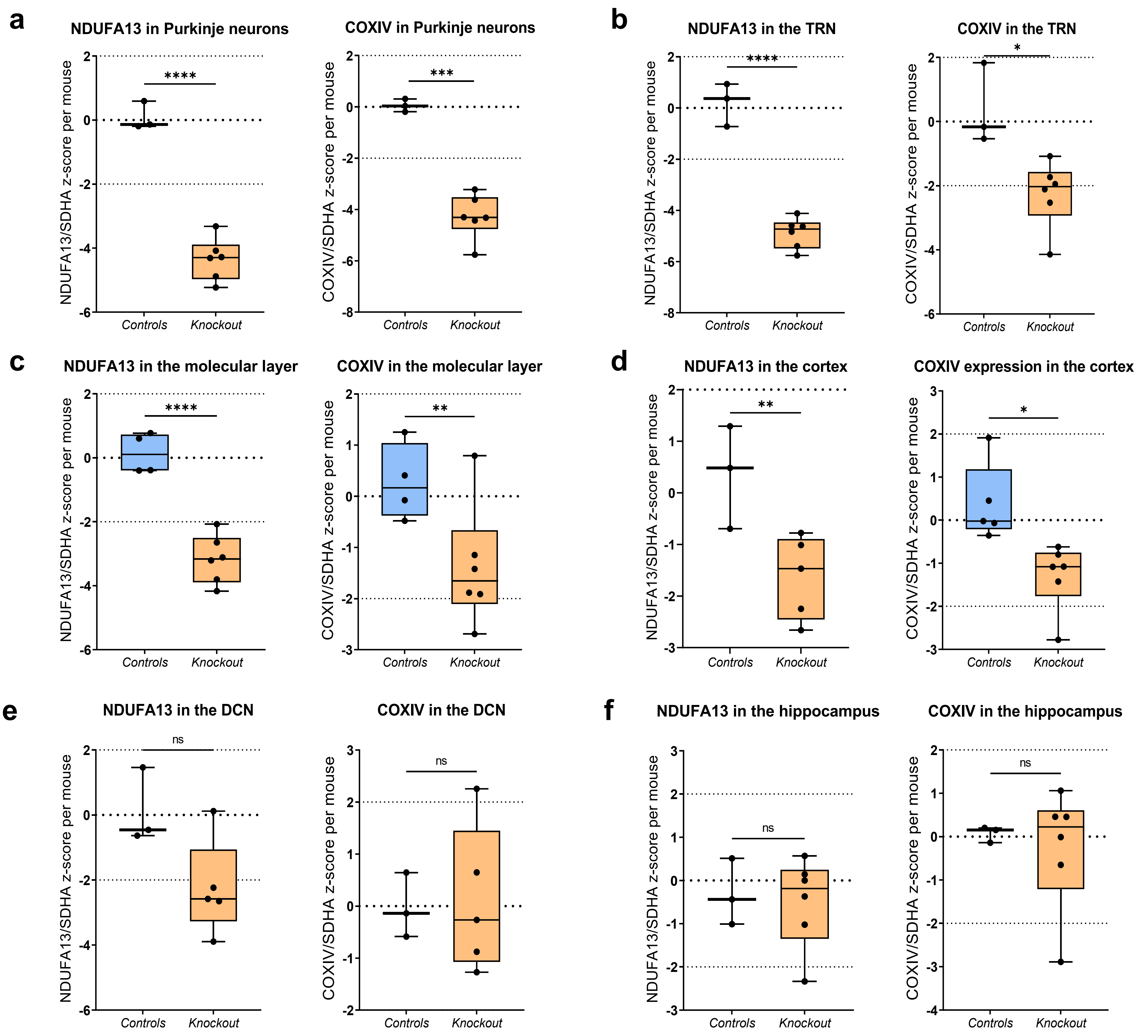

Supplementary Fig. 4: NDUFA13 (complex I) and COXIV (complex IV) expression normalised to SDHA is decreased in PV⁺ cells of *PV*^{cre}*Tfam*$^{-/-}$ mice. a Decrease in NDUFA13/SDHA and COXIV/SDHA z-scores per mouse in Purkinje neurons in the knockout animals (*n* = 276 neurons; 6 mice) *vs.* controls (*n* = 130 neurons from 3 mice) was statistically significant (*P* < 0.0001 and *P* = 0.0001, respectively, linear mixed-effects model). **b** Similarly, in the TRN region, complex I and IV subunits were significantly reduced in the knockout group (*n* = 282 neurons; 6 mice) *vs.* controls (*n* = 119 neurons; 3 mice) (*P* < 0.0001 and *P* = 0.0123, respectively, linear mixed-effects model), **c** PV^{+} neurons of the molecular layer of the cerebellum demonstrated a significant reduction in complex I and IV subunits (*n* = 224 neurons; 6 mice) *vs.* controls (*n* = 129 neurons; 4 mice) (*P* = 0.0001 and *P* = 0.0014, respectively, linear mixed-effects model), **d** PV^{+} neurons in posterior cortical areas demonstrated a decrease in complex I and IV subunits in knockout animals (NDUFA13: *n* = 65 neurons from 5 mice; COXIV: 187 neurons from 6 mice) *vs.* control (NDUFA13: *n* = 33 neurons from 3 mice; COXIV: 97 neurons from 5 mice), which was statistically significant (*P* = 0.0066 and *P* = 0.0105, respectively, linear mixed-effects model). **e** NDUFA13/SDHA expression showed a statistical trend towards reduction in the knockout group in the DCN region (*P* = 0.0589, linear mixed-effects model), whereas COXIV/SDHA expression was unaltered (*P* = 0.8716, linear mixed-effects model; *n* = 77 neurons from 3 control mice and *n* = 119 neurons from 5 knockout mice). **f** NDUFA13/SDHA and COXIV/SDHA expression did not show any differences between the groups in the hippocampus (*P* = 0.9361 and *P* = 0.7388, respectively, linear mixed-effects model; *n* = 55 neurons from 6 knockout mice and *n* = 20 neurons from 3 control mice).

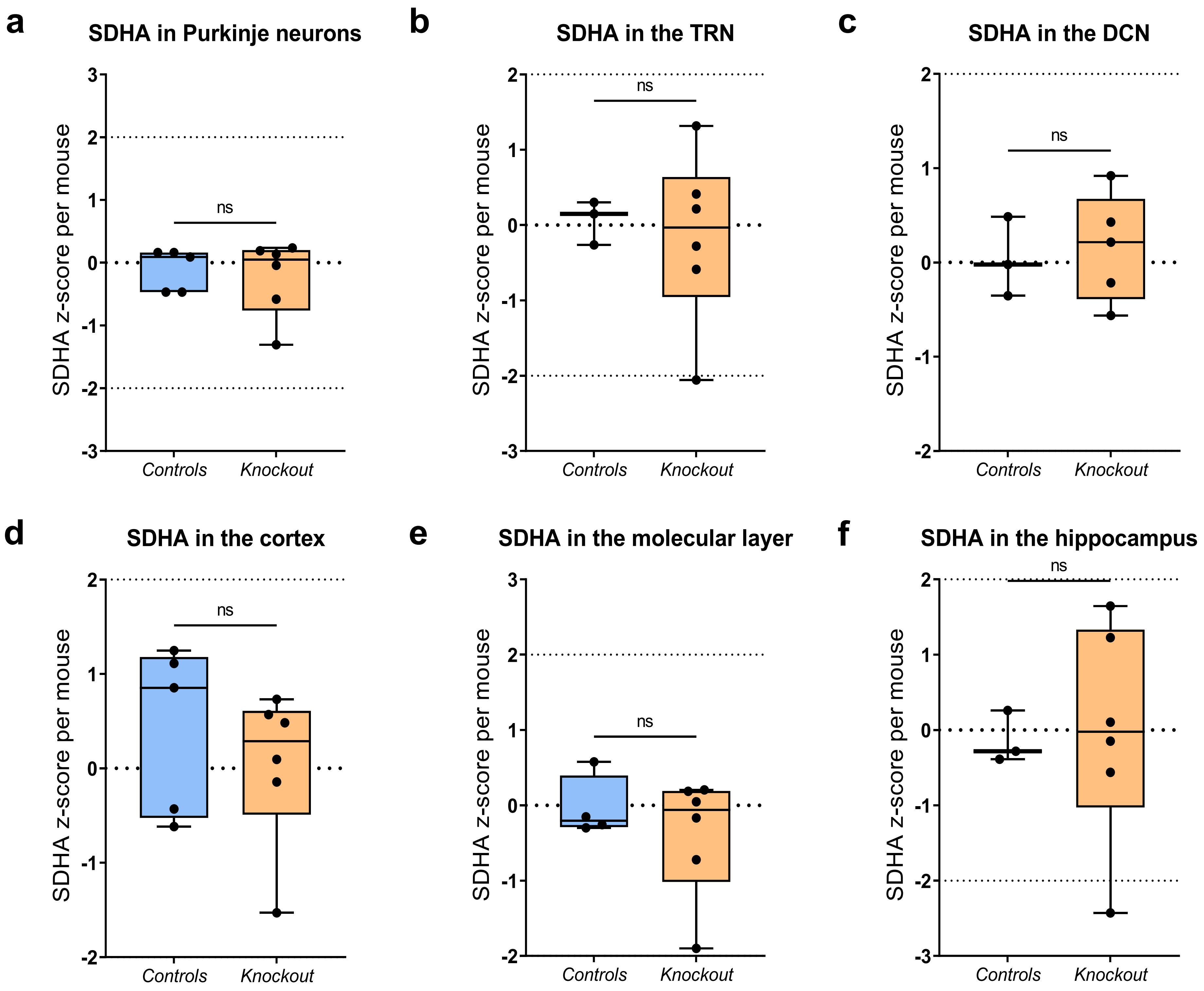

Supplementary Fig. 5: **SDHA (complex II subunit) expression is unaffected in the PV^{+} neurons of knockout mice. a** SDHA z-scores do not differ between control and knockout Purkinje neurons (*P* = 0.473, linear mixed-effects model; *n* = 5 control mice and *n* = 6 knockout mice), **b** PV^{+} neurons of the TRN (*P* = 0.7551, linear mixed-effects model; *n* = 3 control mice and *n* = 6 knockout mice), **c** DCN (*P* = 0.7014, linear mixed-effects model; *n* = 3 control mice and *n* = 5 knockout mice), **d** cortex (*P* = 0.616, linear mixed-effects model; *n* = 5 control mice and *n* = 6 knockout mice), **e** molecular layer of the cerebellum (*P* = 0.473, linear mixed-effects model; *n* = 4 control mice and *n* = 6 knockout mice), **f** hippocampal formation (*P* = 0.8634, linear mixed-effects model; *n* = 3 control mice and *n* = 6 knockout mice),

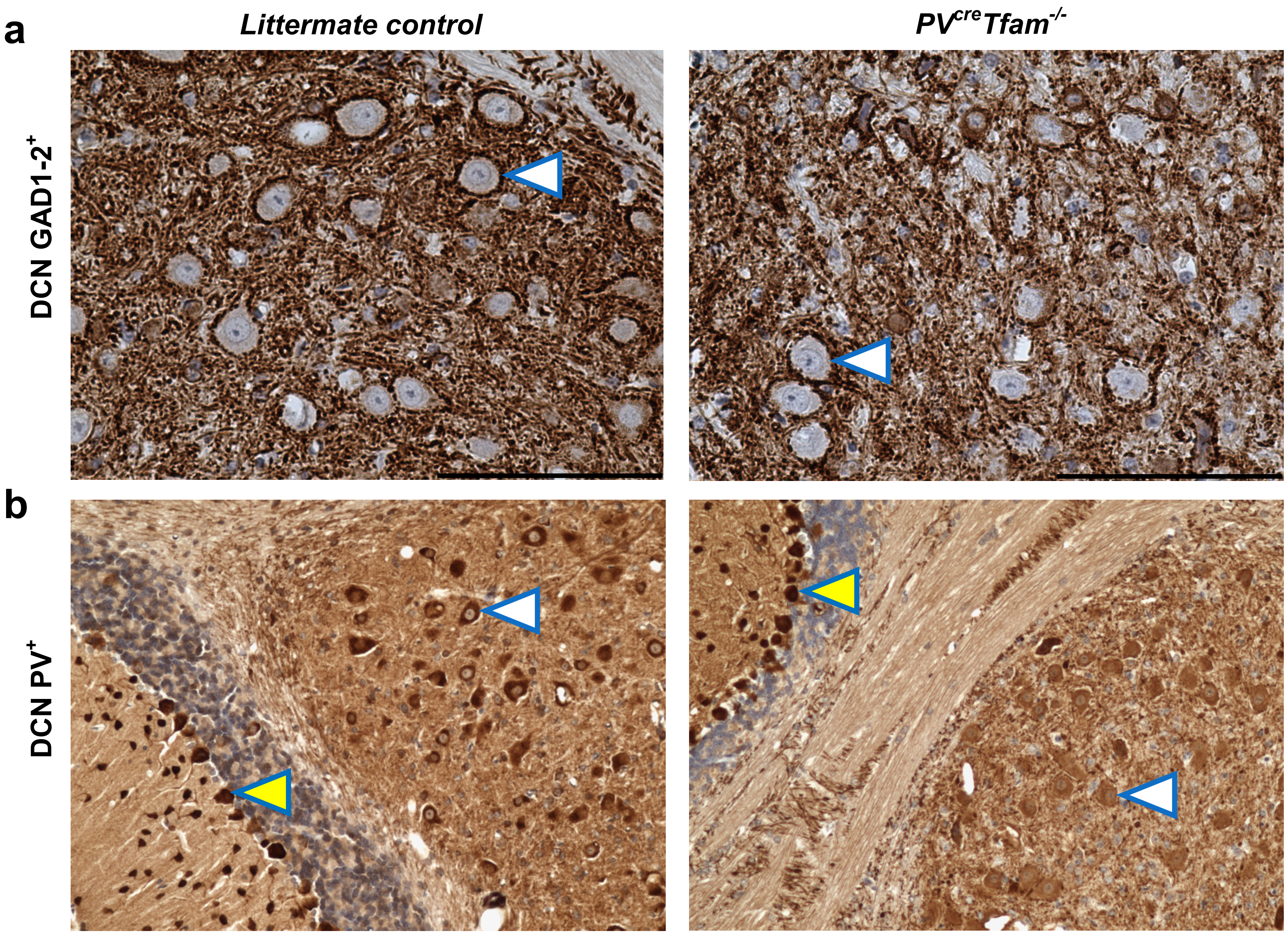

Supplementary Fig. 6: Deep cerebellar nuclei (DCN) neurons are predominantly GAD-negative, but show PV expression. a GAD1-2-immunoreactive signal appears in brown (DAB product) and denotes inhibitory axon terminals and presynaptic bulbs surrounding DCN neurons (white arrowhead). Nuclei are counterstained with haematoxylin and appear blue. Somata of the majority DCN neurons (white arrowhead) are GAD1-2-negative. Scale bars – 100 µm. **b** PV immunohistochemistry with DAB chromogen indicates lower staining intensity in the DCN neurons (white arrowhead) in comparison to neighbouring Purkinje cells (yellow arrowhead) and molecular layer PV$^+$ interneurons, as well as the absence of nucleear PV immunoreactivity in the DCN neurons. Nuclei counterstained with haematoxylin and appear blue.

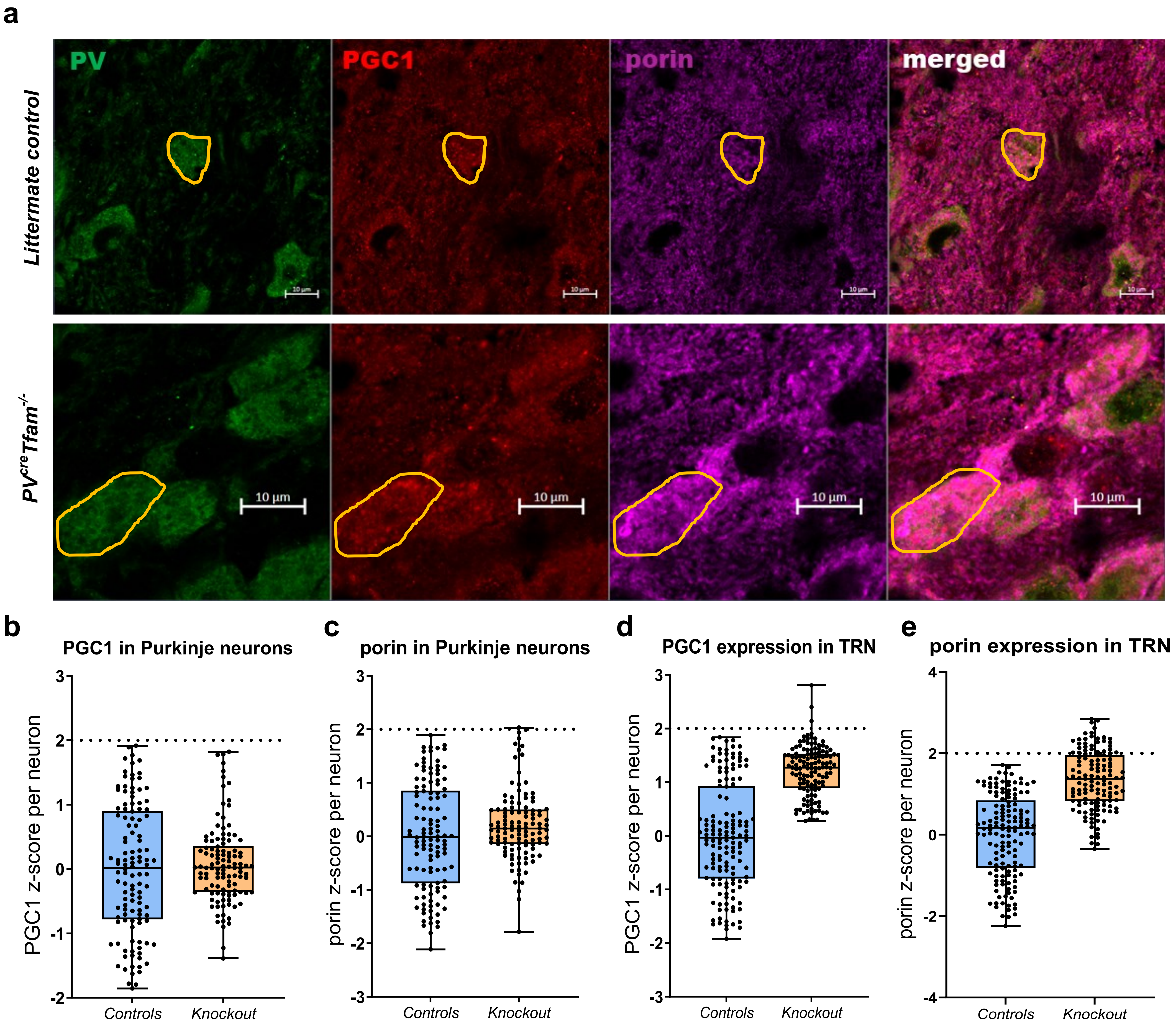

Supplementary Fig. 7: PGC1 expression is not statistically different in Purkinje neurons or PV⁺ neurons of the thalamic reticular nucleus in the knockout mice.

a Immunofluorescence images depicting triplex staining against PV, PGC1 and porin in the TRN neurons. Scale bars – 10 µm. Boxplots and individual data points represent z-scores for **b** PGC1 and **c** porin in Purkinje neurons (*P* = 0.9067 and 0.8362, respectively, linear mixed-effects model analysis; *n* = 118 neurons from 5 control mice and *n* = 114 neurons from 4 knockout mice) and **d** PGC1 and **e** porin in PV^+ neurons of the TRN (*P* = 0.1468 and 0.081, respectively, linear mixed-effects model analysis; *n* = 145 neurons from 4 control mice and *n* = 131 neurons from 3 knockout mice).

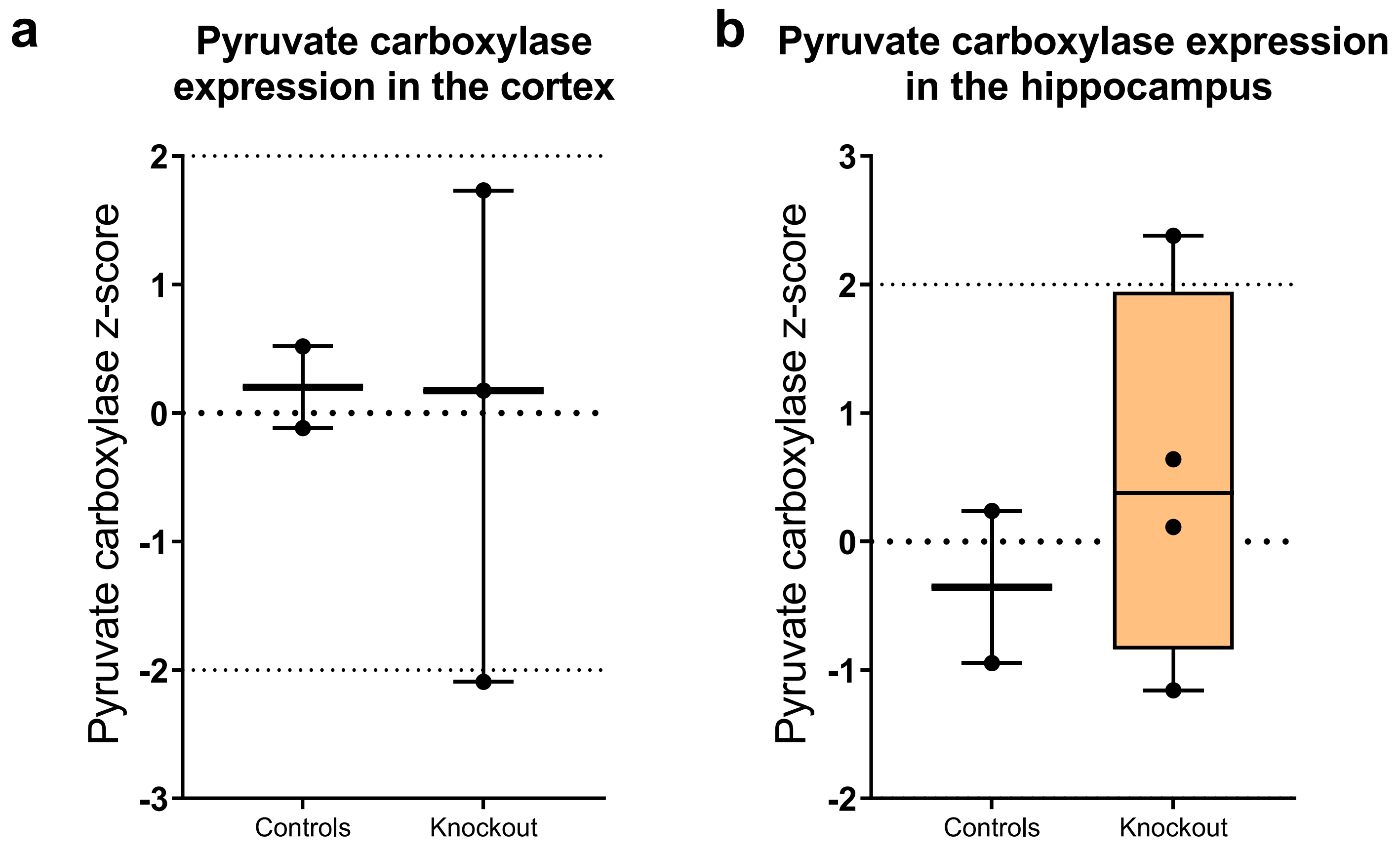

Supplementary Fig. 8: No significant changes in pyruvate carboxylase expression in PV$^+$ neurons in the cortical and hippocampal brain regions of the knockout mice.

a Boxplots demonstrating mean pyruvate carboxylase z-score per mouse in the cortex (*P* = 0.8678, linear mixed-effects model analysis; *n* = 2 control and *n* = 3 knockout mice). **b** Boxplots demonstrating mean pyruvate carboxylase z-score per mouse in the hippocampus (*P* = 0.5173, linear mixed-effects model analysis; *n* = 2 control and *n* = 4 knockout mice).

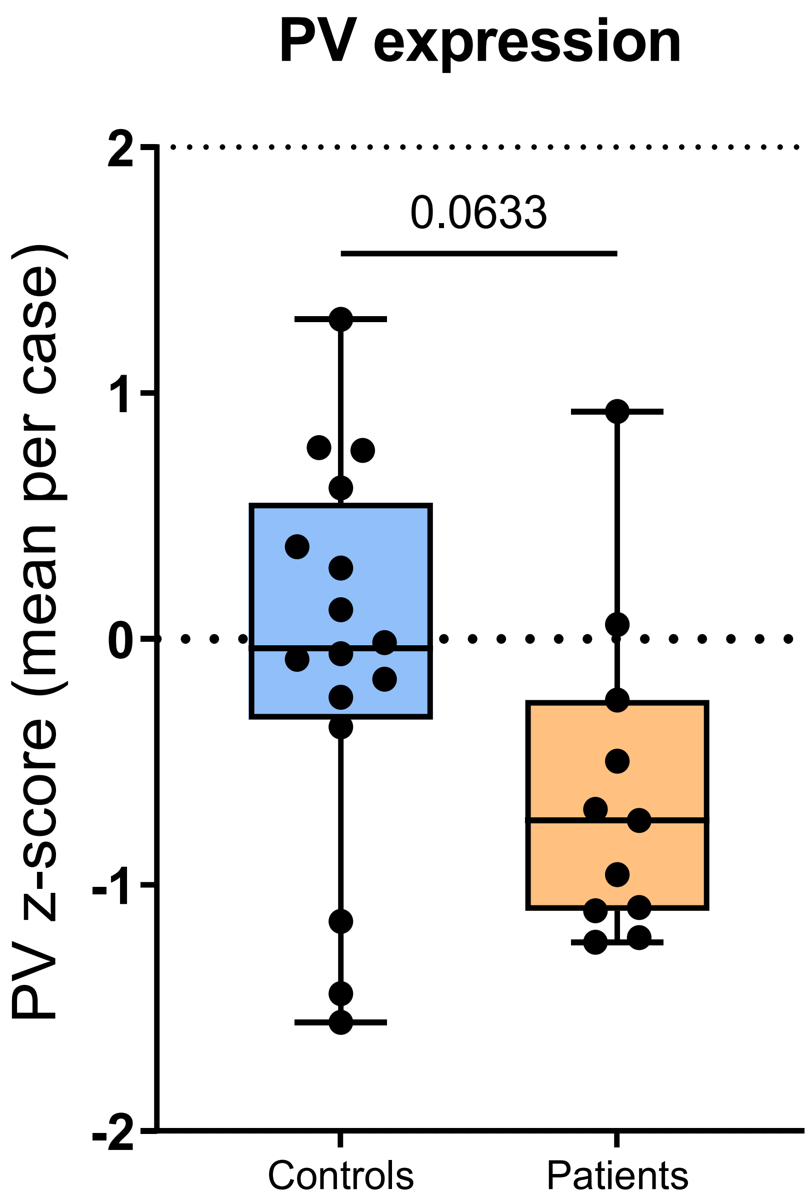

Supplementary Fig. 9: Parvalbumin expression in PV+ interneurons in the occipital lobe in patients with mitochondrial disease. A non-significant decrease in mean parvalbumin expression in PV^+ interneurons in BA17 in the mitochondrial disease group was observed (*P* = 0.0633, *t*-test). In total, *n* = 775 neurons from 16 control subjects and *n* = 469 from 11 patients were analysed.
