# Supplementary material for: A novel mouse model of mitochondrial disease exhibits juvenile-onset severe neurological impairment due to parvalbumin cell mitochondrial dysfunction
Source: Commun Biol. 2023 Oct 23;6:1078. doi: 10.1038/s42003-023-05238-7 (PMC10593770; doi:10.1038/s42003-023-05238-7)
Supplement: Supplementary file 3 — Description of Additional Supplementary Files [file 42003_2023_5238_MOESM3_ESM.pdf]

## Description of Additional Supplementary Files

**File name:** Supplementary Data
**Description:** All source data for figures.
