# Supplementary material for: A novel mouse model of mitochondrial disease exhibits juvenile-onset severe neurological impairment due to parvalbumin cell mitochondrial dysfunction
Source: Commun Biol. 2023 Oct 23;6:1078. doi: 10.1038/s42003-023-05238-7 (PMC10593770; doi:10.1038/s42003-023-05238-7)
Supplement: Supplementary file 4 — Supplementary Data [file 42003_2023_5238_MOESM4_ESM.pdf]

Fig. 1a	Controls	Knockout
	0.72724	0.218341
	-0.00364	0.16539
	0.017547	0.146425
	-0.43283	0.070158
	0.589257	-0.32298
	0.266396	0.091889
	0.47378	0.042556
	-0.0314	0.518006
	0.385212	-0.03523
	0.179812	-0.24516
	0.039435	-0.01279
	0.243665	0.061539
	-0.02868	-0.08047
	0.213646	-0.17491
	0.424143	0.27027
	0.405252	0.092915
	0.48103	-0.0104
	0.412469	0.260628
	0.104293	
	0.460221	
	0.306968	
	0.038112	
	0.389071	
	0.325502	
	0.501	
	-0.61259	
	0.619215	
	0.106294	
	-0.0095	
	0.699495	
	0.184894	
	0.462052	
	-0.42535	
	0.544791	
	0.444659	
	0.327952	
	0.34373	
	0.3803	
	0.17035	

Fig. 1b	Controls	Knockout
	53.3	23.5
	50	35.7
	54.5	26.7
	37.5	40
	36.4	12.5
		25

Fig. 1c

Controls	Knockout
70	60
80	60
70	70
70	100
80	80
	50

Fig. 1d

Controls	Knockout
1195.56	959.25
697.62	823.03
839.49	651.99
399.6	1412.29
1003.32	706.21
840.93	1609.8
284.1	2525.3
466.87	1394.86
582.92	1881.45
958.62	
329.04	
833.23	
1535.23	
956.02	
295.92	
1173.59	
815.28	
851.33	
441.89	
939.15	
1479.9	
431.4	
727.74	
379.1	

Fig. 1e

Controls
0
0
0

Knockout
3.6
1.9
7.75

Fig. 1f

Female controls	Female knockout	Male control	Male knockout
22.8	20.5	23.4	19.5
24.4	19.7	22.3	23.2
21.5	23.5	27.5	22.4
19.8	18.9	24.7	21.3
21.3	16.6	27.9	
	19.5	23.4	

**Fig. 1g**	Controls	Knockout
	158	67
	191.7	58.7
	224	52
	176	47
	204.5	51
	160.5	107
	70	10.5
		22

**Fig. 1h**	Controls	Knockout
	23.33333	12.66667
	27.7	12.3
	31.33333	11.5
	26.5	10
	29.5	11.5
	24	17.5
	13.66667	6
		7.666667

**Fig. 1i**	Days	Controls	Knockout
	77	0	1
	80	0	1
	86	0	1
	89	0	1
	91	0	1
	93	0	1
	94	0	1
	97	0	1
	97	0	1
	99	0	1
	100	0	1
	101	0	0

**Fig. 2b**	Overexpression	Normal	Low	Deficient	Very deficient	
Purkinje	4	3	11	23	62	Purkinje
TRN	0	6	9	12	73	TRN
ML cerebellu	0	23	26	21	30	ML cerebel
Deep cerebe	9	36	16	14	25	Cortex
Cortex	3	70	16	6	5	Deep cereb
Hippocampu	2	76	18	4	0	Hippocamp

Overexpression	Normal	Low	Deficient	Very deficient	**Fig. 2d**
0	5	19	26	51	Purkinje
0	45	31	16	9	TRN
0	62	29	7	2	Cortex
5	75	8	3	10	Hippocamp
24	64	8	2	3	
0	95	4	2	0	

Overexpression	Normal	Low	Deficient	Very deficient	
0	79	19	2	0	Purkinje
0	95	5	0	0	TRN
0	72	19	9	0	Cortex
0	81	19	0	0	Hippocamp

Overexpression	Normal	Low	Deficient	Very deficient	**Fig. 2f**
0	6	30	39	25	Purkinje
0	46	30	13	11	TRN
0	77	21	2	0	Cortex
0	95	0	5	0	Hippocamp

Overexpression	Normal	Low	Deficient	Very deficient
7	84	4	4	1
2	98	0	0	0
0	100	0	0	0
9	91	0	0	0

Fig. 3b

Controls	Knockout
-2.11051	-0.74236
-1.90315	-1.03746
-0.43339	-1.7918
-1.79299	-1.52145
-1.79016	2.247558
-2.00984	-1.8274
-2.09101	-2.49038
-2.19952	-1.92185
-1.33551	-1.78979
-0.57335	-2.61276
-0.48058	-1.36643
-0.64313	-2.40146
-0.21681	-2.3102
-1.09269	-1.03144
-0.20778	-1.85796
-0.59674	-1.00757
-3.60038	-0.68757
-1.42785	-2.79528
-0.88813	-2.78437
-0.69672	-1.90797
-0.80168	-1.4691
-1.04053	-0.29454
-0.96624	-1.29223
-1.68021	-1.39947
-0.63558	-2.56233
0.538212	1.158902
-1.93639	-2.22194
-2.36541	-0.92884
-0.4946	-1.77048
-0.03369	-1.61815
-1.93203	-1.90301
-1.1162	1.444662
-0.64094	-0.84545
-0.75034	-2.28548
-0.62309	-1.7054
-0.81415	-0.27881
-0.93739	-1.75085
-1.52687	1.774817
-1.4399	-0.48845
-1.4867	-0.39194
-1.22687	-2.91096
-0.66702	-2.44925
-1.21276	-1.16943
-1.18064	0.045429
-1.17517	-0.86192
-1.01025	-1.66128
-0.51932	-2.70539
-1.86115	-1.71367
-1.66665	-2.14349

Fig. 3c

Controls	Knockout
0.049534	-1.72694
-0.11967	-1.72467
-0.20408	-1.95695
0.096173	-1.49092
0.055802	-1.90441
-0.08638	-2.11276
0.113626	-2.43189
-0.01145	-1.40139
0.49111	-2.19883
0.274088	-1.94513
0.732983	-2.59444
0.915683	-2.20259
0.451085	-0.61243
0.562248	-2.13992
1.201392	-2.3449
0.525085	-2.11356
0.036216	-0.25674
0.258242	-0.85539
0.251183	-1.13171
0.678458	-1.09821
0.52151	-1.01782
0.067234	-1.00006
0.347223	-0.85397
0.343752	-1.55061
0.416408	-1.31772
-0.16896	-1.31897
-1.77052	-1.049
-1.42855	-0.52215
-0.71294	-0.94893
-0.89792	-0.97902
-0.75472	-0.59864
-0.98037	-0.98602
-1.25716	-0.98862
-0.56853	-0.94045
-1.27229	-0.48088
-1.20034	-1.0639
-1.03624	-1.40023
-1.63925	-1.39051
-1.66053	-2.50203
-0.74115	-2.62589
-0.75082	-2.53966
-0.66297	-2.59591
-1.29766	-2.6952
-1.72764	-1.56836
-1.18999	-1.91279
-0.51705	-2.68061
-0.54422	-1.7424
-1.485	-3.26736
-1.4871	-2.59278

-0.74467	3.283572	0.030575	-1.85358
-1.90497	-0.72897	-1.40536	-2.51153
-0.86436	-0.95345	-0.47333	-2.97642
-1.52492	-1.40326	-1.10571	-2.29123
-1.95466	-2.57714	-1.60021	-3.11318
-0.85714	-2.2187	-0.95788	-2.36252
-1.30814	-2.56792	-0.36241	-2.67193
0.003654	0.378315	-0.10848	-2.87484
-0.81993	-0.78597	0.089248	-2.43926
-0.3503	-1.65273	0.181338	-3.37407
0.037853	-0.90743	0.251882	-3.05444
-1.53486	-1.16568	-0.17453	-2.3328
-0.40479	1.4556	0.277171	-1.66192
-0.61698	-2.27534	0.293081	-2.55182
0.054131	-3.17727	0.683911	-2.78565
0.034977	-0.5499	0.524156	-2.03603
-0.39806	-1.96147	0.530369	-0.92743
-0.29575	1.03138	1.180606	-2.00309
-0.59296	-0.9241	0.108927	-2.04833
-1.04606	-2.4354	0.077073	-1.61659
-0.78476	-1.38828	0.5969	-1.45372
-0.54216	-1.89637	-0.11646	-1.52668
0.563316	0.015662	1.244356	-2.08646
-0.39575	-0.75537	-0.03763	-1.63759
-1.23381	-2.60566	-0.33893	-1.73416
-0.13753	-1.14843	0.146093	-2.28267
-0.42384	-1.21427	0.193451	-1.50624
-0.52101	-0.45203	1.154909	-2.49631
-0.62404	-2.0386	-0.07513	-1.35821
-0.261	-1.14437	0.296069	-2.56949
-0.3463	-0.58467	0.066167	-1.86876
-0.67317	-1.89807	-0.29877	-1.86375
-0.16174	-0.42087	0.300586	-1.7107
-0.55872	-0.0874	0.117429	-2.14891
-0.05505	-2.22379	0.600255	-1.68001
-1.05852	-2.05556	0.643003	-2.11658
-0.54731	-1.63788	-0.18266	-1.29753
-0.65562	-0.86172	0.358893	-1.55155
0.348027	-0.27688	0.027003	-1.04313
-0.18621	-1.40698	0.169579	-0.39475
0.057696	-0.54759	0.389266	-2.53894
-0.15612	-1.75233	0.541551	-1.7934
-0.15519	-1.42615	0.02908	-1.55504
-1.63146	-1.53463	0.272546	-2.55709
-0.09623	-3.79365	0.896074	-2.43259
-0.20017	0.032772	0.48663	-1.88001
-0.16474	1.012564	0.075599	-1.13029
-0.76657	-1.37178	0.453569	-1.69203
-1.0991	-3.26643	-0.08527	-0.99213
-0.6601	-1.64386	1.159452	-1.37343

-1.75668	-3.63777	0.023011	-2.75206
-0.8768	-2.32745	0.375311	-1.73444
-0.90443	-2.16924	0.812067	-1.60893
-0.82688	-0.3938	0.554112	-1.23246
0.117724	-1.27068	0.860573	-0.98353
-1.09684	-2.85858	0.270076	-1.87424
-0.50201	-1.19831	1.545883	-1.55678
-0.32874	-1.38665	0.018181	-1.22237
-1.161	-0.99617	-0.15216	-2.01666
-0.64006	-1.32733	0.562107	-2.3311
-0.24318	-1.90688	1.36074	-1.32122
-0.72007	1.394921	0.507027	-1.4962
-0.11555	-0.14368	-0.11143	-1.08293
-0.35344	-2.98325	0.491687	-1.50915
-0.15352	-1.3119	0.059919	-2.45405
-0.42239	0.410691	0.582886	-2.03945
-0.72801	-1.0585	0.214405	-2.26513
-1.03774	-1.34127	-0.04136	-1.86212
-1.70602	-1.76507	-0.23525	-1.2912
-2.05166	-0.55011	-0.37629	-2.00895
-1.81636	-1.37975	0.449038	-2.08836
-0.63139	-1.59063	0.460791	-1.82154
-0.24412	0.144764	0.016013	-1.77196
0.082988	-2.76796	0.895947	-1.81324
-0.09765	-0.84227	0.159214	-1.79875
-0.0272	-0.35684	0.390755	-1.97979
-0.07183	0.018676	1.324943	-1.89829
-0.07529	-0.38872	0.467342	-0.96843
0.291279	-2.36119	0.133483	-2.23033
-1.81577	-0.75294	-0.11542	-1.67081
0.43279	0.609309	0.156419	-2.15374
-0.41995	0.786672	0.350842	-1.63525
0.028218	-2.0607	0.093731	-2.03013
-0.17485	-0.43277	0.787808	-1.59993
-0.77294	-1.89771	-0.10675	-1.86537
0.42099	-1.62043	1.642392	-1.98483
-0.90457	-1.91541	0.559208	-1.11122
0.257623	-0.04671	0.767555	-0.65795
-0.59861	1.235033	0.67723	-2.18742
-0.47211	0.021334	0.571491	-2.37973
0.379	-0.82057	0.09096	-2.13735
-0.17181	-1.58	0.340957	-2.03448
1.809998	-1.69725	-0.54383	-1.6828
-1.0002	-3.91346	0.22444	-2.0091
-2.3283	-3.02492	0.736403	-1.61481
0.118756	-2.7877	0.258862	-2.47511
-0.17917	-2.34886	0.47642	-1.4652
-0.66928	-0.6284	0.527085	-1.3236
-0.75065	-0.49868	-0.29007	-2.52394
3.746186	-0.96158	-0.39976	-2.15439

-0.4362	-0.27997	0.307311	-1.05939
0.38314	-1.61139	0.559067	-1.64104
-3.15814	-2.14716	-0.32262	-2.14265
-1.94818	-0.42197	-0.34757	-2.18628
-2.64013	-1.35546	-0.47962	-1.44111
-0.30459	-0.26009	0.544465	-2.08741
-0.86123	0.723005	1.373195	-1.12545
-0.39632	-1.85618	0.519506	-1.25372
-0.61233	-1.83568	0.612734	-2.03044
-0.17814	-0.20536	-0.08106	-2.15955
0.045168	-1.3932	-0.00692	-2.24272
-0.37142	-1.53659	0.06904	-0.32734
0.031155	-2.31425	0.01568	-0.79372
-2.23751	-1.05631	-0.07788	-0.72128
-0.66125	-0.5907	0.755049	-0.87412
5.293804	-1.23882	-0.44885	-0.86158
-0.2648	0.001074	0.067234	-0.23516
-0.77237	-2.5112	0.054977	-0.92399
-1.08314	-1.37932	0.421795	-0.75588
0.072352	-2.73831	0.164241	0.07871
-0.43357	-1.60855	0.339295	-0.47304
-0.12053	-3.1905	2.029255	-1.13431
-0.21743	-0.67301	0.030741	-0.97271
-1.1116	-0.31821	0.784243	-0.64611
-1.72937	-1.42566	0.042013	-0.79543
-2.4073	-1.64442	-0.60457	-1.31859
-0.19359	-2.36558	0.722904	-1.81615
0.642573	-1.17641	0.22021	-1.31834
-0.82511	-1.22908	-0.16059	-0.84812
-0.18536	-1.06994	-0.17346	0.296912
-0.32329	-4.61743	0.32505	-1.82066
0.372537	-1.61281	0.617184	-1.14936
-0.302	-0.85308	0.272083	-0.14663
-1.26849	-2.26037	0.226319	-0.13047
-0.36287	-0.86568	0.511049	-1.00563
-0.38856	-3.02686	0.647206	-0.63278
0.253183	-0.7964	0.648996	-1.48211
-0.21242	-1.98055	0.065263	-0.65806
-0.49116	-0.70401	0.095685	0.053823
-0.0674	-0.39985	-0.27145	-0.80344
-0.3794	0.506547	-0.04688	0.37987
0.0022	-1.66358	0.172681	-0.65489
-0.62042	-0.92425	0.473808	-1.40228
0.057267	-0.80889	0.174589	-0.00616
1.152247	0.013244	-0.27774	0.004819
-0.33283	-1.68977	0.589761	-0.00213
-0.67241	-0.62193	-0.04246	-0.59552
0.511716	-1.47991	-0.25005	-0.79553
-0.63256	-0.60833	0.631884	-0.72336
-1.52486	-2.12458	-0.22061	-0.61264

-2.51033	-1.98829	-0.20337	-0.88025
-0.76608	-3.30339	0.103247	-1.4123
-2.01023	-0.10575	-0.39569	-0.98545
4.334188	-1.73815	-0.52245	-0.06495
3.993614	-1.38312	-0.49478	-0.75451
5.770874	-0.85749	-0.4098	-0.75051
-2.56174	-0.98787	-0.44721	-0.81158
-0.79638	-1.95629	0.485907	-1.56068
-0.85046	-1.40004	0.642107	-0.88638
-0.38604	-2.55562	-0.5844	-0.5843
-0.88386	-1.42362	1.18365	-0.94301
-2.24226	0.079817	-0.44779	-0.87391
-3.00855	-2.52313	-0.60638	-1.14082
-0.74184	-0.25463	0.44289	-1.04141
0.640059	1.379626	-0.2458	-0.95598
-0.77757	-0.75335	-0.0144	-1.36594
0.503013	-0.86206	0.214797	-0.69448
-0.65448	0.043372	0.036879	-0.19136
-0.83932	-1.58022	-0.28348	-0.09483
0.468034	-1.64126	0.024425	-1.2036
-0.26696	0.283759	-0.37169	-0.77077
-0.91067	-0.95385	0.206307	-0.90067
-0.986	-0.67968	0.178243	-1.23624
-1.39688	-1.03603	0.099915	-0.93354
-0.10109	0.008738	0.058025	-0.4475
-0.34546	-0.41859	0.664377	-1.0406
-0.32547	-0.47466	0.043088	-0.79778
0.192728	-0.62034	0.674022	-1.1264
0.453337	-1.57261	0.265285	-0.52618
-1.46627	-0.05286	-0.04756	-0.5312
-1.02263	0.653418	0.048543	-1.20686
-0.72366	1.372888	0.338236	-0.9361
-1.38632	-1.08587	0.242864	-1.39908
-1.20539	-2.72687	0.097882	-1.38642
-0.09794	-2.66501	0.26745	-0.75609
-0.4762	-0.44112	-0.29758	-1.0367
-0.18152	-0.6414	-0.48486	0.192898
-1.80501	-0.75816	0.016597	0.036962
-0.00576	-1.44791	-0.07736	-1.02799
-0.33287	-1.39239	0.285866	-1.15768
-0.44924	-0.57309	0.195505	-0.07128
-0.40407	-2.25787	0.335436	-0.88156
-0.81092	-0.61399	0.405756	-0.76147
0.072405	-2.90164	0.804329	-1.05708
-0.47979	-2.78401	0.221621	-0.4155
-0.81862	-1.63095	0.347223	-1.12392
-0.52047	-2.21709	1.485993	-0.85778
-0.54857	-0.71842	0.467051	-1.19311
-1.54608	-0.88932	-0.22384	-0.17355
-0.62896	-0.06428	0.416999	-1.00404

-1.02053	-0.70286	0.506453	-0.79788
-0.64103	-1.55575	0.47555	0.120339
-0.58688	-0.5925	-0.01869	-0.12829
-1.71882	-1.53713	0.058272	-1.10887
-0.08719	-2.42183	0.379944	-0.35466
-0.86478	-2.63657	0.851848	-0.44769
-0.39022	0.389722	0.161609	-0.59753
-0.73303	-3.11679	0.139111	-0.62619
-0.9768	0.079972	1.12913	-1.23356
-0.80366	-0.68991	-0.29327	-0.65908
-0.18484	0.026717	0.571914	-0.69965
0.276206	-0.96903	0.239437	-0.06888
-0.48493	-2.33137	0.644795	-0.47072
-0.21404	-0.91306	-0.14558	-0.74462
0.023949	-0.92428	0.119531	-1.49791
-0.6031	-1.23773	0.278866	-1.36214
-0.53395	-1.59897	0.995945	-0.16412
-1.09119	-0.90142	0.357014	-0.77968
-0.77277	0.759508	0.450793	-0.7868
-0.86422	-0.18833	0.694856	-1.1584
-0.00073	-2.231	0.112655	0.127603
-0.77942	-0.23066	0.489304	-0.95855
-1.59765	-1.73191	0.421869	-0.83535
-0.89054	-1.79917	1.253364	-1.02604
-1.18517	-1.65601	0.138147	-0.73822
-0.814	-2.28598	0.374788	-0.37967
-0.3418	-1.97886	0.22068	-1.48987
-0.35067	-2.09298	0.454737	-0.69727
-0.25867	-1.96997	0.553971	-0.1129
-0.53648	-0.64262	0.505949	-0.59392
-1.41555	2.455274	0.374862	-1.28117
-0.87443	-2.43933	0.425038	-0.75093
-0.26274	-1.69868	0.494573	-0.79927
0.078015	-0.91002	0.18736	-0.11013
-1.52374	-1.61932	-0.25692	-0.16843
-0.69593	-2.66429	0.070517	-0.75619
-0.70123	-0.23846	0.166154	-1.06795
-0.91522	-0.7251	0.482579	-0.74252
-1.16384	-0.83618	0.313946	-1.05788
-1.26717	-1.20002	0.349937	0.112574
-1.46792	-1.59288	-0.10104	-0.8832
-1.10622	-1.76425	0.696892	-0.39484
-0.80642	-2.06079	0.909138	-1.5983
-1.19486	-0.30624	0.697841	-0.43907
-1.01353	-1.60101	0.2067	-0.10156
-0.33733	-2.64689	-0.05923	-1.23953
-0.74306	0.248069	1.493779	-1.21134
0.234109	1.735985	0.719941	-1.55732
-0.55067	-1.0728	0.600255	-0.93365
-1.3418	1.060962	0.800979	-0.8414

-3.03617	-0.80512	-0.27364	-0.49828
-0.57405	-1.41197	0.299668	0.207566
-1.53873	-2.62506	0.854759	-0.72399
-1.96136	-0.96151	0.485835	-0.39211
0.134244	-0.06666	0.122843	-1.30574
0.804831	1.1747	-0.016	-0.59081
-1.23889	-0.92641	0.103328	-0.51558
-1.53178	-2.80723	0.212834	0.347827
-1.33753	1.476164	0.463777	0.679754
-0.91449	-0.70885	0.135655	-0.98161
-0.93871	-2.57505	-0.29565	-1.16531
-1.99004	-1.31387	0.538705	-0.5563
0.024186	0.625449	-0.1702	-0.29813
0.624799	-0.15572	0.043502	-1.04359
-2.64482	0.670055	-0.44472	-1.09109
-0.67499	-1.39934	0.28648	-0.265
-1.73119	-1.28375	-0.24996	-1.21037
-1.23533	-0.76121	0.546027	-0.95094
-3.21045	-2.08438	-0.42216	-0.56554
-2.02246	-1.72561	0.666568	-0.69582
-3.1219	-2.59316	-0.36681	-0.60367
-0.92372	-2.15928	0.099184	-0.96742
-3.28854	-0.08222	-0.44089	-0.1635
-2.8561	-1.05573	-0.24679	-0.92932
-2.16855	-1.42514	-0.15348	-0.22752
-2.80462	-1.81662	-0.59572	-0.97338
-2.26852	-1.63276	-0.46116	-0.69551
-1.53796	-3.41483	1.043425	-1.31172
-0.306	-1.73971	-0.29905	-1.38974
-1.14081	-1.6109	0.226319	-1.07201
-0.9027	-1.8968	0.095034	-0.80858
-0.64782	-1.03205	0.684523	-0.50453
-0.84435	-0.37433	-0.01381	-1.2771
-0.69799	-1.67164	-0.16244	-0.36344
-0.69582	-3.11675	0.21621	-0.28111
-1.61636	-2.88502	-0.15391	-1.35467
-0.60374	0.040553	0.755049	-0.17082
-1.83503	-2.46066	-0.20043	-1.04889
-1.71971	-1.02795	-0.4137	-1.50267
-1.12078	-1.78406	-0.06367	-0.52196
-1.45529	0.479816	-0.37328	-1.09482
-1.77807	-2.14738	-0.13858	-0.27691
-0.79283	-0.66095	0.397001	0.460208
-1.81214	-2.55811	0.23741	-0.06854
-1.25147	-2.85195	-0.31008	-0.69396
-1.94187	-1.52997	-0.18621	0.070599
0.207914	-1.17314	0.469741	-0.54432
-1.3197	-0.04243	-0.14918	-0.05266
-0.57671	-1.15931	0.916063	-0.55283
-0.91021	-0.37251	0.697027	-0.32863

-1.54004	-0.32587	0.159135	-1.34495
-0.94529	-1.27394	0.019181	-0.23085
-0.06957	-0.63259	0.253745	-1.25287
-0.73358	-0.83057	1.092457	-1.53548
-1.91736	-0.1737	-0.08828	-0.70328
-1.11651	1.045557	0.297142	-0.83978
-0.51992	-0.38672	-0.26182	-1.20336
-0.52843	-0.5861	-0.06444	-1.68378
-0.55132	-0.72602	-0.10173	-0.5762
-0.90246	-0.28574	0.5969	-1.47397
-0.97818	-0.98487	0.292927	-0.5778
-1.81947	-0.86125	-0.27509	-1.27192
-0.49957	-1.04955	0.343526	-0.70505
0.292057	-1.96499	0.198347	-0.90475
0.411475	1.289999	0.041434	-0.93989
2.010246	1.957938	0.192898	1.262404
1.599612	0.243206	-0.05003	-1.62275
2.752841	0.062959	-0.14795	-0.44108
0.5337	-0.76048	0.101866	-1.53135
-0.29632	-1.86576	-0.1732	-0.76591
0.563238	-0.69563	-0.29868	-1.0241
-0.74021	-1.7793	0.281637	-1.07816
0.19365	-0.74556	0.524942	-0.50942
2.768575	-1.04646	0.50674	-1.03051
-0.17441	-0.39437	0.280021	-0.66215
-0.13539	-0.80837	0.565497	-0.66359
2.139425	-0.82169	-0.25946	-0.71534
2.328591	-0.1625	0.31021	-1.13892
0.293879	-1.4231	-0.07333	-1.11169
2.363909	-0.98593	-0.17355	-1.19348
0.3119	-1.24205	0.168145	-0.51245
1.451128	0.692511	-0.17682	-0.66636
1.101197	1.468019	0.309142	-0.00557
0.416727	-0.39444	0.608626	-0.15813
0.776063	0.397128	-0.18656	-1.36746
-0.38741	-1.05902	0.097313	-1.02593
-0.41442	0.417779	0.226006	-1.13466
0.651997	-0.94851	0.1537	-0.80504
-0.22189	0.274814	0.4215	-1.50412
1.24491	-0.91659	0.019347	-0.13518
0.751927	-1.47539	0.274628	-0.66523
2.435774	-0.30101	-0.15312	-0.80173
0.625689	-0.13731	-0.06418	-1.07073
-0.02696	-0.37853	-0.19171	-0.28604
-0.01614	-1.14269	0.78827	0.134207
-0.24395	0.903953	0.129054	-0.15664
1.431841	0.279295	0.081981	-1.19203
0.110401	1.681209	-0.15576	-0.10727
-0.63036	0.463415	0.065181	-0.92288
-0.03248	1.328516	0.368124	-1.18818

0.1908	0.661157	0.800782	-0.06555
0.305043	-1.00673	0.068548	-1.00927
2.197142	-0.66996	0.289552	-1.18374
-0.3874	-1.11867	0.014679	-1.03291
1.746591	-3.63893	-2.89051	-1.28488
0.90963	-2.19794	-2.97828	-1.65388
0.749985	0.814776	-3.50225	-0.97091
0.603236	-1.77128	-3.01572	-0.05386
2.016211	-1.76372	-3.34111	-0.57541
0.987707	-2.84473	-3.72909	-1.06436
0.425352	-2.47795	-3.04324	-0.32789
0.653175	-1.12007	-3.22897	-1.17499
0.966776	-2.02835	-3.32453	0.29561
1.058724	-1.19512	-3.37383	-1.39857
0.432306	-3.70912	-3.25361	-1.06783
0.056403	-2.73571	-3.83172	-0.46328
0.157265	-1.47349	-2.69862	-0.4629
0.696585	-2.71572	-3.52658	-0.95923
0.874858	-2.31016	-2.97538	-0.85865
1.938557	1.25385	-2.76232	-0.78021
-0.04389	-2.05631	-2.20373	-1.28575
0.746082	-1.96508	-2.68932	-0.434
1.056785	-1.61106	-3.41467	-1.61905
0.184869	0.623457	-2.85844	-0.75756
-0.1132	-3.68374	-0.04662	-0.98274
-0.15516	-2.24178	0.567332	-0.92488
0.289203	0.125562	-0.10684	-1.08363
-0.42172	1.510213	0.534575	-1.04958
-0.21532	-1.47147	0.921771	-1.05765
-0.16569	-2.51264	0.070435	-1.30213
0.346031	-1.93531	0.177449	-0.6889
0.263196	-1.74941	0.63299	-0.56067
-0.517	-1.5904	0.552766	-0.49614
-0.27704	0.68351	1.23711	-1.1211
1.386681	-2.55371	0.50753	-0.48729
1.068064	-1.64608	-1.51193	-1.28179
1.746067	-2.12005	-0.87795	-0.22241
0.58434	-1.40631	-1.0763	-0.82091
0.142001	-2.35308	-0.79404	-0.4406
0.636108	2.456929	-0.97349	-0.38814
-0.11779	-0.61316	-1.03349	-0.36569
0.293972	-1.89185	-1.30885	-0.89748
2.721005	-2.09202	-1.28216	-1.10172
-0.73623	-2.02148	-1.02696	-0.96787
0.574029	-1.50033	-1.21073	-0.67303
0.9856	-0.3039	-1.28056	-0.3192
0.397103	-0.1095	-0.70733	-0.31699
-0.03191	-1.34949	-0.11325	-0.70028
0.617296	-1.09796	-1.32761	-1.09517
-1.10721	-0.0147	-1.3681	-1.00506

-0.04587	-0.74275		-0.75335	0.021096
-0.53387	-0.16526		-0.09129	-0.36991
-0.25951	-3.27377		-1.02673	-0.59442
-0.66281	2.049418		-0.38381	-0.57002
-0.10228	-0.8784		-0.82963	-0.59974
-0.43013	-1.20056		-0.14033	-1.06888
-0.07987	0.053586		-1.19829	-1.06181
-0.24323	-2.10863		-1.26453	-0.83902
-0.41503	-0.64264		-1.17284	-0.8715
-0.2418	-0.03056		-0.35793	-0.20577
-0.08192	-2.81308		-0.39267	-0.89341
1.052724	-2.87563		-1.26896	-0.66348
0.975168	-2.20219		0.874935	-0.78978
-0.54984	-2.56949		-0.12332	-1.68322
0.107767	-2.7664		0.530725	-0.67786
2.541904	-1.18701		0.173318	-1.19721
-0.57111	-1.71271		-0.40904	-1.68517
0.780905	-2.72394		0.175861	-1.12133
0.338676	-1.64772		1.482681	-0.77575
0.613862	-0.85305		0.280252	-0.8236
0.300648	-3.01693		-0.08785	-1.4829
0.677321	-1.8087		0.400565	-0.62021
0.743108	-1.33125		0.783384	-0.38259
1.712702	0.14658		0.191079	-0.12881
1.473169	-0.2212		-0.07762	-1.34596
-0.11635	0.05639		-0.00532	-1.38897
-0.1292	-1.81791		-0.05616	-1.64559
0.64532	-2.17031		-0.261	-0.46376
1.351784	-1.60782		0.174748	-1.54123
-0.2532	-1.04082		-0.1526	-1.06193
1.9146	1.63497		0.599975	-0.94056
0.24918	0.063751		0.698926	-1.34256
0.057894	-1.2683		0.265672	-1.51074
0.446192	-1.52026		0.193688	-0.91989
-0.29888	-0.69661		0.251882	-1.142
1.861692	0.025875		0.619338	-1.42183
0.289456	-1.99157		0.039861	-0.67683
-0.11082	-1.7495		0.089737	-1.07479
0.020861	-1.58948		0.21974	-0.45837
0.101171	-1.89962		0.117753	-0.63512
0.3104	0.529635		-3.3165	-1.35227
1.825417	-1.48296		-2.92615	-1.78965
0.517776	-1.77566		-3.52732	-1.12982
1.148375	2.933		-3.33281	0.050111
0.744714	-1.01649		-3.03082	-1.57579
1.086793	-1.19354		-3.19345	-1.03051
0.401165	-1.29963		-3.61823	-0.95855
0.669613	0.96633		-2.55145	-0.80793
-0.06679	-1.70384		-2.95848	-1.24674
0.570317	-2.03801		-3.00319	-1.415

1.9794	-0.40672	-3.23165	-0.69231
0.497534	-1.13929	-2.95725	-0.30833
0.464755	-1.03968	-2.50543	-0.77501
1.062225	-2.55763	-2.74763	-0.20952
1.141325	-1.12814	-3.30551	-1.56944
0.146682	-0.7089	-3.22161	-1.20167
0.842753	-0.31491	-2.52214	-0.95486
0.085923	-0.71811	-2.47991	-1.00711
0.325315	-1.07693	-2.00925	-0.70422
1.624495	-1.63862	-0.11498	-0.5764
2.089032	-0.44534	0.118238	-1.02833
0.256402	1.34894	0.412861	-0.0107
1.5644	-0.21776	-0.21954	-0.49604
2.643297	-0.36866	-0.2458	-1.11839
1.312649	-1.77587	0.203235	-1.01006
0.74606	-1.12	-0.25086	-1.02959
1.48135	-0.01355	-0.23876	-1.73757
1.572369	1.017763	-0.21659	-0.63898
1.141729	-1.37135	-0.14322	-1.14509
1.76031	-1.37285	-0.41522	-1.59599
1.497259	-1.94382	0.72324	-0.78191
0.993753	-2.77029	-0.36298	-1.14544
0.676456	-1.69324	0.756382	-1.21715
1.184582	0.172024	0.328996	-0.59201
1.579917	-1.43413	-0.74367	-0.72284
0.795099	-0.40404	-0.764	-1.2338
1.02144	1.360395	-0.24381	-1.41578
1.186132	-0.00943	0.093731	-0.81834
1.233798	-0.95917	-0.28284	-1.02364
0.581748	-1.78195	-0.74851	-0.36775
0.841106	-0.88342	-0.43189	-1.74894
0.272017	-0.87185	0.436656	-0.25204
0.554502	-2.69981	-0.21319	-1.83145
0.890395	-1.49756	-0.54481	-0.96383
1.525879	-1.63263	-0.33707	-0.67426
0.6151	-0.57842	-0.13195	-0.64896
0.794015	-2.4135	-0.58961	-0.31763
0.769927	-1.27245	-0.48943	-0.94904
0.796486	-1.25178	-0.79351	-1.41887
0.71062	-1.60423	-0.01154	-1.14888
2.94323	-2.46843	-0.33345	-1.01896
0.808672	-1.44375	-1.69245	-0.52009
0.500834	-1.91352	-1.26736	-1.52695
2.802216	-1.84618	-0.72222	-1.00426
1.284596	-3.5952	-1.78864	-0.85202
-0.10443	-2.53478	-1.81731	-0.73727
0.976107	-1.0323	-1.62207	-0.576
0.322343	-0.39364	-1.3548	-1.34205
0.741513	0.251669	-0.99201	-0.6554
1.468862	-2.65396	-1.00324	-0.20988

0.667067	-3.14851	-1.46873	-1.57389
1.416306	-1.97369	-1.36632	-0.63044
0.812643	-1.72569	-1.25201	-0.92798
0.840516	-3.03769	-1.00233	-1.85873
0.04964	-2.54514	-1.23112	-0.97586
1.202876	-2.17081	-1.61166	-1.14971
2.014014	-1.62341	-1.11298	-1.18674
0.169775	-2.09744	-0.25801	-0.31745
0.840447	-1.80925	-1.54779	-1.41153
1.36281	-3.04918	-1.34646	-1.47961
0.193019	-3.41068	-1.1131	-1.45164
1.862009	-1.49388	-0.98206	-1.71056
0.937331	-1.41657	-1.15471	-1.08561
0.658977	-2.55175	-0.77141	-0.72243
0.881075	-3.30614	-1.04601	-1.06749
1.311523	-2.01496	-0.55382	-1.02467
0.656199	-1.99217	-1.47646	-1.72481
0.300115	-2.06456	-0.43543	-1.34243
0.570504	-2.44244	-1.59408	-1.1877
-0.1593	-1.84607	-1.1323	-1.10524
1.283113	-1.33798	-1.50968	-1.19227
0.008295	-2.06239	-1.51074	-0.57581
0.763995	-2.19175	-1.02444	-1.00313
1.362503	-1.86774	-0.72587	-0.09569
1.973403	-1.42856	-0.78042	-0.90916
1.378113	-2.18668	-1.07027	-0.5886
1.007384	-2.01241	-0.82285	-0.0931
0.833884	-2.69441	-1.5766	0.156179
0.307353	-2.10758	-0.72524	0.08631
0.635826	-2.74553	-0.85028	-0.01794
-0.09083	-2.00477	-0.03144	-0.39683
1.560499	-1.37613	-0.93365	-0.12654
1.673122	-3.04237	-0.71492	-0.49711
1.580483	-1.27436	-0.54501	-0.1778
2.082114	-1.72864	-1.42532	-0.29905
0.526175	-1.61646	-1.36873	-0.52382
0.408718	-2.75825	-1.00517	-0.19927
1.653889	-2.59171	-0.89528	-0.07917
0.455006	-1.55143	-0.88036	-0.17559
0.813627	-2.2019	-0.7901	-0.00885
1.743227	-1.2938	-1.16698	-0.04374
0.159516	-2.33828	-1.30425	-0.48768
1.173812	-1.02928	-1.17212	-0.76982
0.61071	-2.54104	-1.22735	-0.62122
1.136365	-2.93404	-1.17081	0.012091
0.433122	-1.6086	-1.13738	-0.80654
0.627317	-2.12496	-1.33966	-0.33419
1.708467	-0.66988	-0.71315	-0.93521
1.919915	-2.29486	-1.52083	-0.33883
2.987454	-1.63824	-1.24956	-1.049

0.931478	-1.94254	-1.33036	0.018347
0.866073	-2.87269	-1.72595	-0.18825
1.090423	-0.81645	-1.35151	-0.36008
0.79062	-2.61251	-1.1559	0.071747
0.641824	-2.02471	-1.37432	0.030658
0.370146	-1.27688	-0.54907	-0.54887
0.162417	-1.9652	-1.39524	-0.02519
0.34867	-1.08434	-1.18987	0.657107
1.245708	-1.79519	-0.75609	-0.90144
0.802761	-2.28353	-1.32222	0.157937
1.000853	-2.02341	-0.93588	-0.36569
0.03853	-1.44753	-1.36112	-0.77342
0.93864	-2.33999	-1.419	-0.26463
0.526585	-2.01568	-1.24809	-1.22273
0.691668	0.395082	-1.32109	-0.32521
1.312538	-3.22058	-1.29828	-1.16185
-0.39228	-1.37151	-0.8691	-0.62933
0.452392	-2.65371	-0.86998	-1.50029
1.267252	0.058777	-1.74923	-1.62412
-0.01084	-1.625	-1.21424	-1.68993
-0.11119	-2.07496	-0.73864	-1.74567
-0.16525	-2.44867	-1.28019	-0.72326
0.133377	-1.04527	-1.76508	-1.14675
0.189771	-0.7851	-1.67778	-1.50558
-0.21023	-2.20481	-1.15673	-1.22589
-0.11373	-2.48977	-0.95665	-0.55402
-0.08895	-1.34329	-0.99961	-1.43567
1.010942	-2.29539	-0.59071	-1.61563
1.545641	-2.20448	-0.60981	-1.06679
1.747974	-3.04944	-0.75303	-0.93677
-0.42419	-1.4998	-0.38928	0.018764
1.365131	-1.80406	0.190368	-1.40922
0.963159	-3.69988	-0.71211	-1.41295
1.411593	-1.49625	-0.79639	-0.64071
-0.02406	-2.8111	-0.93599	-1.62316
1.615394	-2.21863	-0.98218	-0.94078
1.020096	-1.88263	-0.07565	-1.28365
0.267684	-2.66392	0.056708	-1.03211
0.731551	-3.26084	-0.94245	-1.19396
0.707411	-1.80431	-0.29602	-0.46811
1.25884	-1.90064	-1.26096	-0.28915
2.921432	-1.57587	-1.22395	-1.88639
1.12854	-2.98798	-1.45203	-1.19613
1.175577	-1.90891	-1.18722	-1.36011
1.251565	-2.50602	-1.30674	-1.46913
1.33373	-2.72646	-1.2676	-1.24736
0.867285	-2.33069	-1.10418	-0.69427
1.169844	-1.363	-0.97259	-1.3413
0.776481	-1.78609	-0.74746	-1.67026
1.297016	-0.02318	-0.80943	-0.82468

1.263779	-2.63682	-1.38081	-1.53562
0.974776	-1.97737	-1.22067	-0.52422
1.142172	-1.2855	-1.37368	-0.63237
1.970178	-2.63863	-0.68931	-0.82124
1.764843	-1.92332	-1.15828	-0.17453
1.112956	-2.32064	-1.73913	-0.16738
0.939655	-2.28468	-1.20312	-0.5825
1.127761	-1.06787	-1.07073	-0.83287
1.056618	-2.0937	-1.47515	-0.66933
0.470637	-2.13937	-1.70212	0.438197
1.724002	-0.7789	-0.93532	-0.23049
1.298657	-1.79097	-1.0467	-0.3253
0.907403	-2.61005	-0.9806	-0.07994
0.710222	-0.03167	-0.75493	-0.7483
0.253795	-2.71974	-1.37305	-0.34944
0.625852	-2.69871	-1.02193	-0.1584
0.168158	0.164236	-0.88145	-0.304
1.200031	-2.36417	-0.95038	-0.73602
2.632025	-2.98663	-1.14888	-0.13483
2.229871	-2.90393	-1.18122	-0.44232
0.499568	-0.53172	-0.9902	-0.79127
0.223618	-2.44185	-1.47279	-0.18098
1.042016	-2.27133	-1.16042	-1.03039
1.445724	-1.62592	-1.76365	-0.76654
0.922981	-2.70017	-0.98387	0.00758
1.074659	-0.75689	-1.39383	-0.47817
0.867588	-4.01565	-1.05165	-1.01223
0.879224	-2.69267	0.027917	-0.46405
1.496075	-0.13337	-0.20908	-0.84281
0.40368	-1.99799	-0.20185	0.479175
1.499551	-1.49986	-0.39324	-0.25149
0.056613	-1.25522	0.094057	0.517
1.149046	-2.33017	-0.1657	-0.07736
0.217185	-1.47014	-0.32465	-0.09371
-0.0925	-3.52268	0.165038	-0.21247
1.338078	-3.33911	-0.20595	-0.04628
0.183185	-3.11374	-0.97102	0.603815
1.849176	-0.24383	0.340353	-0.05761
0.644566	-3.36441	-0.21256	-0.15488
0.756344	-1.21448	-0.50228	0.511694
0.298292	-0.75241	-0.15804	-0.27354
0.65529	-1.95411	0.109495	-0.02198
0.696306	-2.16798	0.016847	-0.01061
0.634593	-0.49971	0.339143	0.83771
1.010181	-2.7052	-0.90199	0.39767
2.93827	-1.89379	0.497385	0.428646
0.598759	-2.28634	-0.72149	0.446624
1.312441	-1.82979	-0.17399	-0.30814
0.025569	-2.27079	0.3652	0.297295
0.415925	-1.1356	-0.07154	-0.35998

0.461954	-2.7213	-0.25955	0.046974
0.656848	-3.32594	-0.52658	0.035553
1.723962	-1.24874	-0.86496	0.743232
-0.02785	-2.81295	-1.00051	0.317296
-0.49484	-4.14723	-0.62477	-0.40203
0.711425	-2.19699	-0.38984	0.20229
0.736942	-2.63029	-0.42722	0.586115
-0.11659	-2.91668	-1.00506	-0.52894
-0.20612	-3.47629	-0.59101	0.422017
0.53623	-2.25882	-0.32734	0.611203
0.110964	-3.43295	-0.43189	0.260178
0.612948	-2.32771	-0.73371	-0.05394
2.348133	-2.40181	-0.81297	0.199451
0.070023	-1.16547	-0.42493	0.369473
0.160497	-3.02231	-0.68621	-0.07754
0.30303	-2.34707	-0.7783	0.300662
0.650832	-2.18343	-0.95822	-0.18222
0.60767	-2.09048	-0.72765	0.040441
1.177663	-2.42005	-0.20604	0.604652
0.433456	-1.88521	-0.37009	-0.16464
0.553061	-1.94721	-0.59291	0.488437
1.234072	-1.41747	-0.09396	-0.26454
0.290825	-2.06422	-0.85832	0.067727
3.426602	-1.94769	-0.21623	0.29423
0.354738	-2.24893	-0.48913	0.585133
-0.32173	-0.87646	-0.4156	0.262656
0.214953	-2.46541	-0.2823	0.355509
0.913319	-2.28812	-0.55819	0.580147
0.710667	-2.68725	-0.39267	1.03364
2.685827	-1.83408	-0.57381	1.077685
1.327287	-2.58834	-0.32391	0.280559
0.092695	-2.1239	-0.77968	0.825338
0.530776	-1.06334	-0.06931	-0.19874
0.706709	-0.9211	-0.56873	-0.20497
0.802989	-2.80491	0.099184	0.638449
0.024717	-2.20389	0.216524	0.846537
-0.33588	-1.09493	-0.77968	0.043502
1.30734	-2.48784	-0.27564	-0.04781
0.243696	-1.38441	-0.74126	0.228899
0.446593	-3.04633	0.001136	0.424301
0.526713	-1.91846	-0.27282	0.174828
1.568607	-1.6723	-0.30419	0.27023
-0.63767	-1.92909	-0.74472	0.02018
2.301873	-2.30916	-0.62254	0.634857
0.030207	-2.68618	-0.1149	1.026406
1.012569	-2.55194	-0.73801	0.639623
-0.28309	-1.699	-0.07351	-0.15963
0.843648	-2.427	-0.29886	0.316687
1.799812	-1.65563	-0.27109	0.311735
1.805899	-2.00861	-0.75451	-0.32697

0.542912	-2.55573	-0.22671	0.265749
-0.39779	-3.03411	0.618157	0.666363
-0.57676	-2.74903	1.017248	0.15098
-0.78498	-2.40007	0.333316	0.131792
-0.10665	-2.15788	0.025756	0.408718
-0.16294	-2.71014	0.586536	0.213855
-0.83096	-2.11814	0.268763	-0.02603
-0.07039	-2.24753	0.849647	0.140798
-0.6118	-2.33522	0.642658	-0.26209
-0.34137	-2.79302	0.905002	-0.02781
-0.2064	-1.27683	1.301255	0.947457
-0.33529	-0.44885	-0.08312	0.877376
-0.4015	-1.56129	0.813443	0.677708
-0.2727	-2.97878	0.1549	0.311888
-0.59617	-2.89175	1.083421	0.335134
-0.61595	-1.55378	0.484533	0.918982
0.628497	-2.54085	1.452508	0.564861
-0.56856	-2.11458	0.202999	0.307921
-0.87057	-2.95274	0.029163	1.13353
-0.85154	-3.44853	0.568531	0.05679
-0.7323	-1.48196	0.350766	-1.23222
-0.6284	-2.48845	0.34058	-1.11862
-0.36611	-2.26652	0.626483	-1.64366
1.416628	-2.96588	0.636999	-0.80408
-0.55232	0.263653	0.244265	-0.86017
-0.27556	-1.75389	-0.32262	-0.4156
-1.07672	-3.62054	0.217466	-1.75793
-0.35361	0.438324	1.152429	-1.34533
-1.04696	-2.0225	0.301963	-0.89462
-0.33543	-0.15415	0.281868	-1.30089
-0.61787	-2.06921	0.857021	-0.92454
-0.8836	-0.78449	0.680913	-0.4656
-0.1799	-2.00915	0.70123	-1.44241
-0.03763	-1.56822	1.285633	-0.7934
-0.72332	-2.71459	1.668106	-0.72222
-0.4012	-1.47364	0.388447	-1.17607
0.156895	-1.66642	0.874357	-1.68937
0.099284	-1.00628	0.214797	-1.17715
-0.14692	-2.71933	0.225145	-1.48855
1.34715	-1.22611	0.618018	-0.36279
-0.15594	-1.50447	0.728821	-1.09424
-0.41478	-1.06877	0.405089	-1.71352
1.082779	-2.3565	0.866635	-1.05004
0.106041	-1.85895	1.423907	-0.49994
0.002366	-0.56568	0.465087	-0.92765
-0.37718	-2.95497	1.253249	-0.94123
-0.66586	-1.39248	0.826186	-1.50055
-0.74313	-2.16986	0.071091	-0.68436
-0.08773	-2.31191	0.725864	-0.34981
0.234338	-1.81301	0.873392	-0.78935

0.384232	-2.81695	0.83719	-0.65734
-0.74154	-1.21104	0.869082	-1.24809
0.363177	-2.03518	1.770763	-0.6891
-0.27768	-1.93686	1.040431	-1.12463
0.059281	-3.48801	1.287392	-1.25912
0.030848	-2.71594	0.858442	0.536926
-0.32282	-2.77372	0.923101	0.750047
0.519372	1.408399	1.874593	1.095945
-0.37116	-2.52089	0.988503	-0.24598
1.938207	-2.6875	0.023344	0.125425
0.227859	-2.49447	0.221699	0.850424
0.343502	-1.36667	1.026713	0.106332
0.491316	-2.41788	0.950661	1.162457
0.159706	-2.15378	0.448453	0.71043
0.137001	-1.88161	0.743232	0.349108
8.65E-05	-1.33165	0.853401	0.629807
-1.12044	-2.10219	0.352348	0.72324
-0.55284	-2.16442	0.639968	1.722071
0.765414	-1.92939	1.858468	1.117031
-0.5103	-1.78838	1.549289	0.410495
-0.54923	-1.36588	0.084596	0.93164
-0.02031	-2.49718	0.786356	1.071878
2.439994	0.222391	-0.02046	0.698113
-0.96956	-0.99941	0.407608	0.621212
-0.56734	-1.93578	0.930502	0.013427
0.114278	-2.24676	1.02057	0.225927
-0.4614	-3.05213	0.872621	1.036456
-0.27863	-1.66366	0.035387	0.62579
-0.72089	-1.78777	0.364525	0.517358
0.040415	-1.18899	0.702382	-0.0462
0.453986	-1.06259	0.885906	0.751115
-1.2595	-1.33998	0.90462	0.555883
-0.35588	-3.23494	0.915302	0.282868
-0.03939	-1.29625	1.370486	-0.29501
-0.66183	-2.79103	0.240528	-0.05658
0.188147	-2.96358	0.209533	0.541266
-0.74557	-2.82127	0.948462	0.231635
-0.75424	-3.38488	1.015093	1.034498
-0.42708	-1.79583	0.851848	0.049121
0.062944	-2.0342	0.188627	0.913079
-0.21036	-2.82309	0.075599	-0.33688
0.282638	-3.28437	0.984713	0.214012
-0.71021	-2.82975	0.326948	-0.35205
0.047278	-1.15671	0.803935	0.999041
-1.10454	-1.78498	0.853724	0.532437
-1.50161	-2.49292	0.149218	-0.43333
-0.54771	-2.80405	0.853401	-0.21435
-0.41499	-1.65554	0.464942	-0.22501
-0.27321	-2.81092	1.517872	0.274551
-0.43236	-2.11731	0.57853	0.034807

-0.62288	-1.99197	-0.07325	0.225458
-0.33138	-1.9368	0.571773	-0.09552
0.437864	-4.32678	0.21464	0.074698
-0.80713	-1.23718	0.794269	0.57649
-0.16798	-1.75209	0.651473	1.340882
-0.43529	-1.40525	1.59542	0.192661
-0.47839	-1.04543	0.762239	1.107882
-1.32566	-2.38606	1.779273	0.389266
-0.79183	0.229013	0.773922	-0.24147
-0.76343	-1.36594	0.312574	0.587658
-0.33183	-0.0073	0.305172	0.755582
-0.98394	-2.68867	1.281944	0.533221
-0.01275	-2.32375	0.418402	0.655665
-0.3653	-1.67858	0.872621	0.94997
2.228175	-1.04884	0.580006	1.100629
-0.68664	-0.54235	0.122681	1.398127
-0.74541	-2.53646	0.716572	0.679549
-0.33624	-1.34754	1.031619	0.495583
0.257139	-2.77286	0.759911	-0.1136
-0.58267	-1.46153	0.695399	0.316002
-0.90808	-2.03109	0.15154	1.262861
-0.71784	-2.66887	0.05539	0.179275
0.031324	-2.18243	0.971135	0.566767
-0.72394	-1.48288	0.790117	0.45751
-0.43167	-2.13989	1.021676	-0.1289
-0.31898	-1.69191	0.322392	-0.25231
-0.66445	0.063917	0.606256	1.258802
-0.54956	-1.71821	0.874357	0.383527
-0.67793	-1.68097	0.861799	0.957813
-0.74626	-1.30312	0.967825	0.783847
0.179689	-1.40646	0.361973	-0.07634
-0.63664	-2.15338	1.28183	0.600813
-0.70505	-3.61316	0.789589	0.029163
-0.53394	-2.07092	0.184034	0.332938
-0.43453	-1.52461	-0.19349	1.333342
-0.27998	-0.92193	0.358668	0.495727
-0.42952	-2.76262	0.30395	0.465524
-0.42722	-2.19367	0.572619	0.586045
-0.1242	-2.65807	-0.31551	0.694381
-0.1489	-2.0674	-0.04144	0.771934
-0.20671	-1.42419	0.149859	0.09894
-0.65981	-2.1073	0.012258	0.974441
-1.84742	-1.73424	0.757581	1.018663
-0.63086	-1.06007	0.545459	0.215896
-0.57334	-1.23657	0.676957	0.809577
0.026434	-2.61592	1.399224	0.611481
-0.23619	-1.24739	1.589062	0.843878
-0.44918	-2.12158	1.167106	1.345398
-0.98346	-1.54048	0.797231	0.348958
-0.34135	-1.527	1.792143	0.839724

-0.80475	0.330757	0.975439	-0.32438
-0.77838	-2.14978	1.023765	1.266458
-0.92866	-1.84437	0.421427	-0.07325
-1.23945	-0.82376	0.91346	0.579936
-0.27702	-2.03111	0.65491	0.582676
0.260166	-2.17246	1.043058	0.884432
-0.37442	-2.22755	-0.05778	-0.08329
-1.23378	-1.11812	0.102922	-0.27965
-0.85981	-2.07758	0.44201	-0.14199
-0.85109	-2.29818	0.961947	0.093079
-0.97325	-0.73052	0.823576	-0.77543
-0.72118	-0.73461	1.070365	-0.36073
-0.69829	-1.16374	0.926203	-0.71471
-0.91988	-1.28634	0.248775	0.003397
-0.25288	-2.28494	0.664856	0.509398
-1.56798	-1.60369	0.408348	0.288631
-0.35885	-2.37858	-0.17355	0.319883
-0.58716	-1.34027	0.61489	-0.70494
-1.17583	-2.11792	0.63216	-0.26118
-0.46258	1.073868	0.828207	0.080591
-0.81612	-1.72915	0.912444	-0.3715
-0.63162	-2.48863	0.423417	-0.17992
-0.85129	-1.9695	0.634857	-1.97078
-1.48112	-2.21201	-0.2242	-1.59422
-0.91917	-2.32399	0.516857	-1.3418
-1.11454	-1.77943	-0.11021	-1.06054
0.617518	-2.12895	-0.39003	-2.09769
-0.55208	-1.89313	-0.219	-1.95043
-0.74338	-1.84249	1.208833	-2.30975
-0.12348	-2.32246	-0.18346	-2.48364
-0.91653	-2.37074	-0.03356	-1.57592
-1.3184	-1.74283	1.403004	-2.47795
-0.90801	-3.13719	1.207149	-1.72991
-1.32111	-2.09342	0.600883	-2.29291
-0.99517	-1.44446	1.964788	-2.45793
-0.43531	-3.17306	0.447868	-2.35002
-0.67222	-2.37299	1.156503	-2.33671
-0.10221	-1.66497	1.012752	-0.96753
-0.85432	-1.66084	1.394122	-2.01543
-1.30445	-2.56671	0.761241	-1.83613
-1.14209	-1.2916	0.598997	-1.62179
-1.16824	-2.97216	0.961258	-2.05317
0.769502	-2.23656	0.900158	-2.04522
-0.6841	-2.61783	0.282868	-2.56602
0.167857	-1.86434	-0.16368	-2.16617
0.883905	-1.70717	0.639002	-2.444
-0.04401	-1.09638	1.529484	-1.68029
-0.7355	-2.65457	0.369623	-1.73048
-0.14769	-2.00129	-0.08578	-1.71916
-0.14646	-2.0251	1.471975	-1.69735

-0.85415	-2.44596	0.58099	-1.77196
-0.1265	-1.47875	0.750514	-2.12121
-0.13279	-0.81892	0.899265	-2.11388
0.09786	-1.04305	1.155441	-2.07056
-0.5878	-1.65392	0.448526	-2.36304
-0.78166	-2.85029	-0.04934	-1.54605
-1.51985	-2.03038	0.162885	-1.94136
-0.12745	-2.0704	0.600464	-1.94937
-0.11679	-2.03075	0.988565	-2.09262
-0.2401	-1.60993	0.556237	-2.26797
0.846552	-1.7843	0.123892	-2.45616
-0.74307	-2.35331	0.531011	-1.7859
-0.45227	-2.82618	0.50185	-2.13399
-0.0217	-2.34292	1.038475	-1.47188
-0.50802	-2.22214	1.162987	-1.86404
-0.62357	-1.26973	0.629669	-2.06428
-0.0471	-2.19409	0.836539	-2.44981
-0.568	-1.61683	1.584246	-1.63195
-0.32327	-2.17148	0.539488	-1.46808
-0.27971	-1.81748	0.19511	-2.06805
-1.10686	-2.21233	1.351577	-2.15874
-2.21591	-1.64259	-0.44376	-1.94423
-0.52897	-1.14229	0.205992	-1.6561
0.062978	-1.10472	0.564367	-2.37077
-1.02199	-2.76195	0.514922	-1.32297
-0.15073	-1.457	0.738882	-1.79543
-0.50578	-2.26936	0.360997	-2.54111
-0.58894	-1.95705	-0.13195	-2.31043
-0.63205	-1.54402	1.550964	-2.39131
-0.64839	1.174647	1.483376	-1.64932
-0.66471	-2.87757	0.816649	-2.05317
-0.14661	-2.01612	1.314499	-1.40216
-0.84206	-2.77122	0.771072	-1.80584
-0.77075	-2.80555	0.747577	-1.90232
0.026949	-2.93056	1.032783	-1.6835
-0.68475	-1.91257	0.192344	-1.59177
-0.82971	-2.08849	1.211736	-1.85108
-1.02825	-1.78928	-0.02764	-2.39754
0.43514	-1.79959	0.6691	-1.86021
-0.39942	-1.64433	0.523798	-1.56593
-0.53909	-1.99624	0.812067	-2.49166
-1.26136	-2.91166	0.593822	-1.98269
-0.2658	-0.09875	0.858055	-2.30182
-1.30523	-0.06443	-0.1519	-1.90337
-0.5564	-0.93091	1.010039	-2.38007
-0.84179	1.566677	-0.14182	-2.08694
-0.8193	-2.17089	0.973568	-1.93381
-0.30452	-1.32888	0.253589	-1.76866
0.006283	-2.2837	0.805708	-2.60439
0.089979	1.108602	1.158803	-1.54243

0.510184	3.424355	0.647069	-1.8913
-0.05973	-1.47959	0.213855	-0.7462
-0.09592	-1.66133	-0.03254	-2.52412
-0.82	-1.28675	0.409089	-2.76251
-0.78979	0.188265	1.659303	-2.03168
-0.32804	-2.87488	0.799599	-2.13767
-0.42024	-1.1469	-0.34395	-1.66636
0.350087	-2.03505	0.342619	-1.5983
-0.10074	-2.22601	1.002814	-2.05208
-0.68672	-1.84853	0.417664	-1.73601
-0.53512	-1.30295	0.017931	-1.89442
1.59823	-2.83764	0.331271	-2.19475
1.558979	-0.29234	-0.24354	-1.43709
-0.57009	-0.55071	0.98011	-1.84243
-0.57487	-2.14864	0.583167	-1.72892
0.322785	-1.9509	0.53429	-2.34609
0.046838	0.541902	-0.41151	-2.0351
-0.03586	-1.46884	0.865926	-2.34934
-0.93148	-2.03183	-0.17143	-2.38906
-0.78268	-2.32588	0.230853	-1.94786
-1.1545	-1.66543	0.559138	-2.57974
-0.12093	-0.56256	1.328081	-1.78446
0.727203	-0.44855	0.741225	-3.57581
0.141824	-1.29007	0.726469	-3.43956
0.168337	-1.4426	0.346394	-3.54103
-0.03269	-1.39415	1.04983	-3.30643
-1.19539	-2.45553	0.340731	-2.95437
0.234841	-2.88332	1.157506	-3.30163
0.484001	-1.09466	0.132678	-3.03082
0.301757	-0.77206	1.786056	-3.6658
1.104066	-1.32076	0.43247	-3.10693
1.392463	-1.32713	1.843221	-2.18189
0.212845	-1.79588	0.807807	-3.69395
0.082729	-1.6219	0.731641	-3.3785
0.972575	-1.66397	0.018681	-2.99674
0.613149	-1.3087	1.302046	-3.35662
-1.81734	-2.84382	0.26884	-3.70502
0.520209	-0.37838	-0.11039	-4.08276
0.435268	-1.59619	-0.17965	-3.40995
-0.12052	-1.49328	0.289705	-3.77486
-0.22392	-1.93267	1.281148	-3.37267
0.634764	-2.31657	0.679686	-2.49774
1.243284	-1.12929	0.248853	-2.31584
-0.28378	-1.73725	0.417295	-3.18001
0.64635	-2.37769	1.78781	-1.8735
-0.42502	0.332943	0.756782	-2.37956
-0.00599	-2.83116	-0.13308	-2.44348
0.327693	-3.40303	0.114598	-1.96181
0.316958	-3.0837	-0.33725	-1.70184
0.621865	-2.83178	-0.22617	-1.83511

0.096031	-2.32057	-0.50854	-2.48863
2.454791	-1.81862	-0.51578	-2.12025
1.106138	-0.88379	0.505446	-2.33178
-0.29346	-1.29723	1.262918	-1.92809
0.348317	-2.78746	0.376283	-1.41153
0.93717	-2.51363	0.715965	-1.7292
-0.10909	-2.32548	0.704074	-3.00569
0.620702	-1.69227	-0.06974	-3.44956
0.305241	-1.86014	-0.10995	-3.3543
0.053344	-1.33165	0.970573	-3.0107
-0.07187	-2.18043	1.181133	-3.14718
0.059889	-2.62755	1.395714	-3.62276
-0.24896	-2.17362	1.427224	-2.92737
-0.18804	-1.91867	0.642934	-3.56121
-0.2772	-2.58197	1.178086	-4.03253
0.412032	-1.88564	0.238268	-2.79718
0.487504	-2.80199	1.173452	-3.38716
-0.0997	-2.44922	-0.0994	-3.34712
0.336313	-2.10496	1.864375	-3.07632
0.238452	-1.81479	0.859669	-1.67666
-0.27188	-1.47254	0.284943	-1.34747
-0.94895	-1.66119	-0.18958	-0.74304
0.545808	-0.21188	0.891793	-0.88572
-0.38967	-2.20329	0.062551	-0.56524
-1.35455	-2.68205	-0.10113	-0.61112
0.311332	-2.32385	0.934796	-1.3091
0.454368	-1.48764	0.864637	-0.48952
-0.52307	-1.23139	0.201186	-1.05708
4.038694	-3.17069	0.256692	-0.33744
0.056719	-3.26064	0.826707	-1.18074
0.382385	-3.19215	0.664514	-1.18578
-1.11395	-2.84655	0.749446	-1.61741
2.12165	-0.1271	0.252969	-1.23612
0.213616	-1.41185	-0.07513	-0.9042
-0.59732	-2.39635	0.318894	-0.27282
-0.82951	-1.63546	-0.15989	-0.87412
-0.42512	-2.12432	-0.27892	-1.37979
-0.07669	-3.12948	0.749113	-0.8999
0.075684	-1.55054	-0.11316	-1.05685
0.180404	-1.18026	1.581602	-0.83546
0.545054	-3.32358	0.549647	-1.07549
0.580248	-3.56681	0.206307	-1.80729
0.191449	-0.77012	0.561612	-1.14758
0.385384	-1.8125	0.348204	-0.55859
-0.06575	-2.9567	-0.30768	-0.51823
0.237766	-2.3906	-0.20622	-1.66053
-0.2144	-2.2688	-0.40213	-0.97395
-0.31388	-3.4993	1.257772	-0.99269
-0.2104	-0.69611	0.728753	-0.26827
0.03307	-2.86961	0.720952	-0.77596

0.496449	-1.60157
2.847449	-2.16935
-0.11845	-1.25516
-0.84036	-2.32911
-0.56479	-0.02744
0.359907	-3.3091
0.10477	-1.11797
0.393653	-1.729
0.410324	-2.53657
-0.16805	-0.67731
0.28218	-3.54148
3.728881	-3.67554
0.655871	-3.10577
-0.3777	-2.00865
-0.34187	-1.27782
0.470463	-2.95451
-0.61045	-1.96119
-0.40998	-2.29247
0.910485	-2.89878
-0.16801	-2.83833
0.321472	-1.88791
1.228534	-1.9399
-0.808	-2.10614
1.040817	-3.73622
-0.54474	-2.57389
0.266652	-3.16263
-0.3074	-3.03506
-0.06089	-1.8901
-0.66396	0.740269
0.299799	-2.56738
2.253006	-1.00671
0.435735	-3.59118
-0.11534	-2.90878
0.091506	-0.52608
0.744138	-1.04722
0.211141	-2.59291
-1.03407	0.495648
-0.97692	-2.38414
-1.06712	-2.04547
-0.5578	-1.68491
-0.50675	-2.8978
-0.83311	-2.62565
-1.1168	-3.20478
-0.6875	-2.36735
0.580622	0.009155
0.130773	-2.6619
-1.02926	-2.89313
-0.98994	-3.11309
-1.36645	-3.56357
-1.40674	-2.52136

0.607093	-0.15848
0.10552	0.383974
0.262656	-0.63725
0.902963	-0.56893
0.108765	-0.15242
1.276429	-0.81372
0.700078	-0.32142
1.047574	-0.2985
1.277283	0.213384
0.662801	-0.48069
1.20145	-0.80451
3.079558	-0.17294
0.790249	-0.64336
1.990635	-0.17532
0.470395	-0.15339
1.375238	-0.15207
-0.08432	-0.54996
0.730902	-0.60175
0.749446	-0.14357
0.479682	-0.00557
-0.2233	-0.88803
0.943871	-0.62568
0.182528	-1.17691
1.123891	-0.62274
0.463704	-1.22711
0.565426	-0.978
0.444062	-1.03131
1.603364	-1.50253
-0.11056	-1.55141
0.753116	-1.24222
0.39202	-0.98477
0.261959	-1.16865
0.966888	-1.03028
0.617462	-0.77459
0.957563	-1.11498
1.649766	-0.7621
1.428528	-1.05846
0.74771	-0.86333
1.003679	-1.76465
1.092999	-0.68477
2.094661	-1.12039
0.374339	-1.16328
1.185113	-1.55866
0.217779	-1.11709
1.722519	-1.40832
0.265517	-1.08969
0.368049	-1.36315
0.661224	-0.95508
0.560905	-1.75279
1.529695	-0.9841

-0.27973	-3.1322	2.068637	-0.44904
-0.8952	-3.0154	0.13139	-0.37911
-0.3085	-2.09221	0.232806	-0.61597
-0.81005	-2.56467	0.477943	-0.96338
-0.51909	-1.52927	0.723846	-1.09191
-0.8625	-0.5144	1.119001	-0.72806
-1.54055	-1.87156	0.48757	-1.08759
-0.97123	0.292183	-0.12742	-0.75978
-0.08277	-2.91409	0.119046	-0.51274
-1.12179	-2.55327	0.845759	-1.22419
-0.78224	-0.62599	0.766293	-0.33818
-1.19791	-2.98038	-0.11386	-1.2282
-0.82975	-2.63774	0.294307	-1.00847
-0.79908	-1.47483	0.278096	-0.8207
-0.8036	-2.82902	0.3646	-1.32761
-1.43041	-1.43026	0.009753	-1.00086
-0.70041	-2.15324	0.918157	-1.25679
-0.36543	-1.81952	1.15957	-0.61496
-1.30023	-3.00456	0.620518	-0.66841
-0.81049	-0.84799	0.948085	-0.6269
-0.81792	-1.49697	0.932397	-1.53735
-0.6974	-1.07043	0.723106	-0.90938
0.013567	-2.89644	0.435849	-0.83935
-0.98447	-0.63582	0.774585	-0.80248
-0.03685	-0.47413	1.54011	-1.29468
-1.15509	-1.99238	1.45116	-0.76823
-0.02193	-0.94716	1.180371	-1.19239
-0.93064	-1.1964	1.459997	-1.37763
-0.49708	-1.47949	1.556299	-1.34861
-1.37267	-0.73053	0.662732	-1.58404
-1.09516	-1.49122	0.621351	-1.57146
-1.09181	-2.65625	1.289943	-1.40601
0.829967	-3.41804	0.829966	-0.55888
-0.65697	-2.80118	1.666489	-1.3369
-1.21151	-1.89815	0.231869	-1.71155
-0.54195	0.324275	0.445892	-1.08375
-0.88234	-1.44589	1.269196	-1.11181
-1.10683	-0.02616	0.635133	-1.06066
-0.97724	-1.83083	1.110576	-1.22541
-1.32732	-0.46869	0.410421	-1.22298
-1.43005	-1.08665	0.893391	-1.25287
-0.70705	-1.84277	0.745973	-0.57481
-0.87291	-2.67548	1.048062	-0.60679
2.241593	-1.00745	1.112549	-1.59408
1.916171	-2.89579	1.207788	-1.34546
-0.77067	-3.52751	1.191365	-0.87708
-0.85475	-0.90762	-0.03136	-0.97958
-0.96432	-2.74386	0.828598	-0.85995
0.125043	-1.78163	1.25365	-0.94323
-1.5296	-0.03708	0.48663	-1.22079

-0.23923	-2.38942	0.741359	-1.1817
-0.61803	-1.91399	1.63112	-0.64529
-1.0325	-1.94903	0.307082	-1.33966
0.416775	-1.98347	0.357239	-1.52708
-0.02925	-1.3062	0.99297	-0.90442
-0.52117	-0.49255	1.535222	-1.47659
-0.0333	-2.05073	0.428866	-1.055
-0.91689	-1.96242	0.933597	-1.37635
-1.44856	-1.36875	0.20851	-0.92599
1.41399	-1.2535	1.404372	-1.44202
-0.86198	1.077352	1.228808	-0.94837
-0.71553	-1.91578	0.731507	-1.24552
-0.46074	-2.32709	0.604024	-0.98014
-0.73241	-0.62639	1.553162	-1.21098
0.086519	-2.69771	0.473373	-0.65335
-1.16533	-2.2391	1.903178	-0.44395
-1.60208	-0.6783	0.597669	-1.0763
-0.52183	-0.18845	1.167341	-0.91845
-0.45623	-2.56738	-0.03051	-0.86278
-0.11897	-3.04104	0.590811	-0.61799
-1.19092	0.072357	0.784838	0.055719
-0.92027	-2.96861	0.751449	-0.85854
-1.52187	-1.28831	1.117329	-1.19769
-1.4844	-1.39843	0.322164	-0.98093
-1.03939	-1.3589	0.745372	-0.9815
-0.9207	-0.07523	0.292851	-0.83935
-1.28046	-3.0619	0.332862	-1.02021
-1.43197	-1.30774	0.470032	-0.68807
-1.37747	-1.2094	0.449842	-0.87839
-0.25734	-1.6031	0.443769	-1.22918
-1.19978	-0.52596	0.595012	-1.54177
-1.16536	-1.88312	0.550072	-1.32986
-1.2264	-2.8731	0.455832	-0.84162
-1.23436	0.683839	-0.24959	-1.63401
-0.21557	-0.91408	0.825272	-0.81662
-1.12519	-1.59671	1.396866	-1.26158
-1.36918	-2.14734	0.320643	-0.83589
-1.10392	-1.95502	0.222953	-1.20977
-1.17057	-1.18515	0.759046	-1.26613
-2.07212	-1.71371	-0.00826	-1.0225
-1.15383	-3.15616	0.81593	-1.59232
-0.87083	-2.04122	1.006891	-0.89495
0.308983	0.440273	-0.16174	-1.05719
0.766428	-2.13732	-0.94078	-0.7462
1.17589	-2.0205	-0.59743	-0.85321
1.319147	-0.90727	-0.651	-1.38094
0.943461	-1.99635	-0.5769	-0.85778
0.759288	-2.11302	-0.11498	-1.01896
0.578998	-0.92553	-0.39626	-1.60429
1.292854	-0.9638	-0.44223	-0.96001

1.013634	-0.97511	-0.26182	-1.08433
0.920838	-1.60685	-0.50854	-1.58228
0.107979	-0.96571	-0.76105	-0.61294
2.069091	-2.97504	0.266986	-1.32736
0.776491	-0.71209	-0.13169	-1.52748
0.85132	-1.84114	-1.03188	-1.12098
0.635163	-2.70863	-0.04671	-0.83902
1.148609	-2.99914	-1.48697	-1.45594
0.112172	-2.51293	-1.52642	-0.98184
0.028435	-0.86786	-1.61235	-1.20759
1.126344	-2.84722	-1.69819	-0.80194
0.309078	-3.62149	-1.38285	-1.33916
0.591891	-0.35943	-0.40383	-1.15518
0.553305	-1.15024	-1.23953	-0.96181
0.23414	-1.58344	-0.43228	-1.33011
0.204523	-2.94735	-1.47109	-0.37056
0.768831	-2.4057	-1.7859	-0.86616
0.127373	-1.77262	-1.57227	-1.49857
-0.30139	-2.14846	-1.72609	0.043337
-0.23081	-1.33986	-1.721	-1.50306
-0.3591	-1.91222	-1.10864	-1.06008
0.306524	-0.43712	-1.67207	-0.78637
0.428881	-3.83535	-0.63126	-1.16531
0.279295	-1.21878	-0.61092	-1.17344
0.014005	-1.79942	-0.99666	-0.96843
1.008458	-2.41993	0.933787	-1.37445
0.117165	-2.58186	-0.15172	-1.02387
0.042783	-2.29534	0.224049	-0.87784
0.682603	-2.33603	-0.27546	-1.43528
-0.14172	-2.98075	-0.35084	-0.05232
1.029593	-1.11669	0.566838	-0.92077
1.050296	0.214354	-0.0389	-1.43955
-0.03556	-1.78259	0.403458	-0.96225
-0.07446	-1.21519	1.280182	-1.50359
0.201037	-1.58045	0.180386	-1.06424
2.763496	-2.7097	0.447648	-0.50072
-0.29711	-2.37925	0.29193	-0.56335
1.80669	-1.78304	-0.06751	-0.86224
0.098194	-1.86865	1.19947	-1.27969
0.151495	-1.59992	0.448965	-0.89847
1.440771	-2.32881	0.246132	-1.37852
0.102513	-2.56058	0.79973	-1.41204
1.472636	-2.24825	-0.26999	-1.42364
-0.28552	-2.9192	0.003062	-0.84909
0.127756	-1.06186	0.527728	-1.08072
-0.25355	-2.88812	0.328845	-0.94826
-0.8365	-2.01894	-0.30217	-1.3553
-0.47254	-2.21922	-0.43428	-1.21279
0.491114	-2.03249	2.430783	-1.04854
-0.04018	-1.71592	1.323261	-1.06378

0.136108	-2.00724	-0.20025	-0.85387
-0.10074	-0.16282	0.02376	0.054236
-0.01896	1.01824	0.294307	-0.73518
-0.08308	-3.38233	0.613221	-1.26576
1.022878	-3.57767	0.18744	-1.66012
-0.20317	-2.10762	-0.02629	-1.11345
1.335166	-1.8683	0.188785	-0.5866
-0.03523	-2.19057	0.47526	-0.94457
0.763586	-0.95222	0.109414	-1.37343
0.636988	-1.00586	0.489015	-1.23149
1.489038	-1.1047	0.586115	-0.93221
0.856372	1.278422	0.229368	-0.78712
1.9893	-2.75721	0.546524	-1.46965
0.302025	-2.29072	1.621143	-0.75598
-0.07017	-2.2006	0.06707	-0.7065
-0.40839	0.773521	0.076991	-1.0813
0.158912	-1.39615	0.346469	-1.36264
0.524374	-1.14758	-0.05113	-0.57451
0.066993	-2.59937	0.486847	-0.97192
-0.46684	-0.86414	0.121793	-1.08293
-0.15431	1.540286	0.057367	-0.91557
-0.26846	-1.40583	0.066249	-1.09436
0.421341	-2.71972	-0.1116	-1.71775
0.399244	-1.82894	0.119531	-0.79543
-0.62838	-1.56263	0.130987	-1.37801
-0.19456	0.271504	0.359795	-1.11945
-0.1158	-2.03922	0.538136	-0.93777
1.705746	-0.88197	0.202447	-0.88836
1.015418	2.256673	0.345865	-1.55598
0.574368	-0.42844	0.623363	-1.17799
2.288069	-1.33705	0.642107	-0.764
1.588253	-1.8479	0.279867	-0.84476
0.503805	-2.88147	0.077236	-0.30226
2.166657	-1.83626	1.018355	-0.84432
0.131215	-2.41986	0.82964	-0.87817
0.597851	-0.35665	1.301538	0.120339
0.569679	-2.04	0.416925	-0.41569
0.156217	-1.09016	0.76782	-1.2282
0.21014	-2.74053	-0.16227	-0.46724
-0.06069	-1.39336	0.101866	-0.93132
-0.13038	-1.27622	1.118046	-0.75051
1.227576	-2.06747	0.080427	-0.55363
1.303329	-2.06979	-0.04986	-1.1439
1.692067	-2.56996	0.641762	-0.91026
0.363737	-2.49501	0.369997	-0.54047
0.214008	-1.4101	0.327859	-0.94591
0.960775	-2.61774	0.451524	-1.18086
-0.09919	-0.80188	0.301963	-0.72869
0.256335	0.919767	0.750514	-1.15102
1.641756	-2.66926	0.789194	-1.06494

-0.15629	-0.24092	0.502282	-0.71867
-0.37301	-1.62589	-0.23049	-1.33011
0.107007	-1.7026	0.241852	-1.09623
1.134674	-1.29535	0.02118	-0.76654
0.558868	-2.49676	0.17292	-0.93889
0.634276	-2.3149	0.635133	-1.06471
-0.57539	-0.22469	-0.12158	-0.89924
0.691418	-0.77003	0.131148	-1.43269
1.178481	0.100328	0.474389	-0.76834
0.323738	0.034645	0.00666	-0.5859
0.379278	-0.17066	-0.11264	-0.53041
0.589148	-2.90646	-0.21667	-0.4975
-0.18211	-2.06627	0.353402	-0.33893
0.491979	-1.94246	-0.01053	-0.94078
1.808981	-1.54989	0.036216	-0.93655
-0.72978	-1.65372	0.084024	-0.99722
0.070825	-0.46773	0.042427	-1.16507
0.982025	-0.15928	0.117187	-0.96731
-0.29651	0.055912	-0.34953	-0.81211
0.681398	-1.99615	0.338463	-0.18914
-0.08635	-0.17892	0.101297	-0.67118
-0.02041	-1.19478	-0.28148	-0.8715
-0.57862	-2.71459	-0.21292	-0.59081
0.004154	-3.06709	0.316307	-1.70283
-0.17825	1.966535	0.195505	-0.77331
0.255098	-2.35434	-0.13361	-0.83308
0.081183	0.396576	-0.27892	-0.95755
0.020591	-2.90324	0.552837	-1.35
1.646685	-1.48196	0.249863	-0.19447
0.630109	-2.6032	0.46516	-0.90464
1.011258	-2.24795	0.282637	-1.05615
0.162424	-1.95097	-0.10407	-0.62082
-0.17007	-1.05395	-0.08269	-1.36898
0.28229	-1.45421	0.936563	-0.8999
0.576175	-2.78369	0.929618	-3.81434
0.015191	-2.25507	-0.29987	-3.46343
1.150784	-1.90246	-0.42817	-3.7226
0.460479	-2.19289	0.057202	-3.57854
-0.36108	-2.53461	0.389713	-3.69986
0.461071	-1.0304	-0.23237	-3.66732
-0.05277	-0.30243	0.131068	-3.39865
0.566896	-1.97816	-0.0894	-3.67933
-0.00572	-2.29689	0.090716	-3.24261
0.821882	-1.85911	0.317525	-2.98262
-0.05212	-2.22196	0.056872	-3.91009
0.309172	-2.21868	0.087616	-3.22674
2.907387	-2.88993	3.68006	-4.05224
0.648258	-1.3515	0.315241	-2.92167
0.579832	-2.58781	0.611272	-3.6883
0.676012	-2.87478	-0.585	-3.32821

-0.45871	-2.28319	-0.07265	-3.2462
0.538589	-2.8441	-0.42312	-3.83413
1.183905	-1.5909	-0.76274	-3.42484
-0.10478	-2.54478	-0.27272	-3.1244
0.571005	-2.84366	-0.74063	-2.72246
-0.10729	-1.757	-0.99507	-3.69138
-0.34593	-1.93382	0.120985	-3.19588
1.151317	-3.29154	-0.72556	-3.65994
0.751394	-0.28558	-0.49945	-3.91696
-0.17411	-2.0707	-1.00426	-3.06462
-0.35939	-2.96221	0.28156	-3.45841
-0.64708	-2.66089	-1.06436	-3.18705
0.451729	-2.35503	-0.35681	-3.64168
0.315615	-2.49008	-0.4043	-3.16071
0.827757	-0.64327	-0.6951	-3.33051
-0.90135	-2.71914	-3.13718	-3.28683
0.15042	-2.67455	-3.75752	-3.1203
-1.19264	-2.70022	-3.51878	-3.9046
0.466263	-0.97256	-3.72883	-3.59945
-0.69138	-2.66893	-3.61472	-3.17517
-0.37196	-1.29023	-3.63384	-3.12786
-1.52392	-2.01075	-3.76119	-3.77987
-0.41301	-2.97797	-3.2712	-3.65308
-0.33371	-2.14064	-3.46439	-3.16312
-1.06642	-3.46433	-3.51829	-3.1908
-0.72617	-0.98957	-2.80031	-3.50783
-0.53054	0.385363	-3.75412	-3.2755
-0.18346	-2.00023	-3.38037	-3.72545
-0.57913	-0.5287	-3.64877	-3.37944
-1.04962	0.147648	-3.12202	-3.77776
-2.35462	-2.13573	-4.00305	-3.48725
-0.24338	-3.35688	-3.72805	-3.81834
-0.40826	-3.04553	-3.82556	-3.40712
-0.16542	-2.47799	-3.41231	-3.56442
-0.7052	-2.30756	-3.90159	-3.41916
0.578489	-2.351	-3.90872	-3.12592
-1.66583	-2.77023	-3.78647	-3.66376
1.004592	-2.22589	-3.96265	-3.18859
-0.55357	-3.50161	-3.38131	-3.2176
-0.06464	0.917154	-3.67831	-3.44003
-1.34511	-0.34455	-3.355	-3.17737
-0.88382	-1.77822	-3.34065	-2.95889
-0.03025	-2.8439	-3.90323	-3.91283
0.344235	-2.82958	0.177052	-3.10844
0.037897	-0.55025	0.139111	-3.65562
0.824312	-2.2856	0.009586	-3.41207
1.328733	-1.11605	0.651679	-3.13415
-0.41487	-1.34819	-0.36625	-3.26691
-0.57689	-1.58669	-0.14479	-3.61096
0.628773	-1.61996	-0.12594	-3.4776

-0.06271	-2.36994
-0.00012	-1.93273
1.419247	-1.19652
-0.40331	-1.87531
0.108058	-1.98566
0.543064	-2.60113
0.707203	-2.71651
0.271141	-2.58456
-0.74374	-0.77513
0.363304	-1.19431
0.41376	-1.18317
1.316429	-0.48989
0.313869	-0.04852
0.767671	-2.83873
-0.01658	-1.81624
0.124495	-1.64908
2.330138	0.169911
0.159932	-2.7561
0.287782	-2.66343
0.584996	-0.30546
0.056772	-1.22598
-0.44516	-2.39741
0.214545	-1.09707
1.488333	-1.91737
0.142376	-1.94215
0.600256	-1.02529
0.553466	-2.20871
1.239672	-2.49053
-0.09352	-1.9853
0.53014	-3.85362
1.130163	-2.18376
1.029327	-3.90685
0.263094	-1.13266
0.764166	-2.51748
-0.36108	-1.22662
0.511643	0.890001
-0.48645	-1.97376
0.610913	-1.38791
-0.28452	-2.35356
-1.2475	-2.4513
0.962906	-2.08621
-0.39763	-2.07698
0.071252	-3.14354
0.167746	-0.7719
1.37999	-2.61713
0.764603	-1.62573
1.166794	-1.60917
-0.18584	-2.93268
0.150506	-1.56083
1.17885	-0.61963

0.318514	-3.14675
-0.1839	-3.90625
0.407238	-3.27188
-0.41855	-3.27007
0.245898	-3.2973
0.136057	-2.93941
0.377404	-2.90221
-0.01423	-2.63258
0.180941	-3.6032
0.296989	-3.56838
0.37718	-3.401
-0.12115	-2.95951
0.212598	-3.16355
0.228196	-3.82449
0.581973	-3.46919
0.790117	-3.49401
1.527112	-2.66741
-0.20141	-3.07121
-0.74063	-3.87868
0.18229	-3.12138
-0.19589	-2.8334
-0.23597	-3.74549
-0.07017	-2.93125
-0.33475	-3.16268
-0.92366	-3.06547
-0.47914	-3.07078
-0.35298	-3.38271
-0.14891	-3.42365
0.474461	-3.26172
-0.09336	-4.07236
1.218054	-3.16049
-0.82102	-3.49885
-0.33558	-3.32292
-0.75367	-3.50176
-0.45077	-3.18705
-0.48447	-3.25834
-0.65233	-2.65351
0.792161	-2.57425
0.05712	-3.39677
-0.66943	-3.5768
1.172395	-3.30757
-1.02387	-3.12332
-0.32234	-2.43487
0.269999	-2.84332
-0.65397	-3.63586
-0.34218	-3.35314
0.10552	-2.84908
0.354531	-3.59795
0.812591	-3.0555
0.183954	-3.24912

-0.04928	-0.00552	1.60357	-3.20561
-0.06328	-2.773	0.718998	-3.34597
-0.85688	-3.01169	-0.07994	-2.76716
0.348061	-3.10337	0.49897	-3.79786
0.222325	-0.86584	0.457364	-3.76539
1.33467	-1.81082	0.23507	-3.26781
0.329058	-1.78228	0.097882	-3.81568
2.196487	-2.42494	-0.13727	-3.61798
0.092151	-2.3219	0.343903	-3.39912
0.298236	-3.17792	0.884753	-2.68875
0.908201	-1.35504	0.427468	-3.1149
0.751359	-1.15378	0.225614	-3.79627
0.933812	-2.56056	0.482289	-3.01468
-0.20621	0.157032	0.081572	-2.69235
0.922817	-1.57566	1.074843	-3.23813
-0.06443	-3.50159	-0.18346	-3.27867
1.344921	-2.89588	-0.14444	-2.81308
0.664527	-1.3364	0.610228	-3.06038
0.417788	-1.65768	0.043585	-3.02305
1.390986	-3.24918	0.073551	-1.88268
-0.00366	-2.32597	1.064545	-3.1961
0.035279	-3.52821	0.490532	-2.98159
0.591701	-2.75354	0.124215	-2.93063
0.211445	-2.09825	0.438271	-3.20096
0.250586	-2.29907	0.404941	-2.69273
1.152192	-2.31866	0.281329	-3.21516
0.50588	0.461594	0.035553	-3.38809
0.31404	-1.85857	0.343677	-3.14914
0.802178	-2.64319	-0.01971	-2.87123
-0.41261	-2.07932	-0.04	-3.28116
0.925703	-3.70274	0.146975	-3.3729
-0.32273	-2.70333	0.238346	-3.26127
-0.22926	-0.83288	0.379496	-3.08763
0.40797	-2.02885	-0.41398	-2.90464
0.044459	-2.21946	0.299438	-2.86843
1.373841	-2.55136	0.531225	-2.57498
0.586988	-2.76075	1.105606	-2.39373
-0.0576	-2.4775	0.67566	-3.30734
-0.03643	-1.37257	-0.20248	-3.19323
0.806425	-1.72591	0.613569	-3.30004
0.939802	-2.1345	0.558147	-3.33465
0.408856	-1.64636	0.486269	-3.55776
0.846559	-1.10147	0.167827	-3.53098
2.390191	-3.09247	0.793873	-3.42816
0.012119	-3.12204	0.394549	-2.90525
0.73564	-1.26883	0.494068	-3.5105
-0.22329	-2.48512	0.16121	-3.25834
0.458313	-0.84941	0.738547	-3.48387
0.824717	-1.88697	0.27432	-3.43528
0.255859	-0.89681	0.425996	-3.99825

0.698932	-2.37106
1.180815	-1.89438
0.700902	-2.79745
-0.10341	-2.20389
1.459622	-3.65953
-0.08641	-2.53189
0.855078	-2.07054
1.786955	-1.46973
2.261123	-2.26425
1.152358	-1.30073
0.265666	-1.42714
0.525203	-1.88524
1.684331	-2.66236
0.375194	-2.55483
1.262148	-1.97047
-0.20949	-1.58267
0.13671	-2.74746
-0.90915	-2.41133
0.24741	-0.17919
0.847582	-0.02216
1.010588	-2.75732
-0.25876	-2.77114
-0.33531	-2.32967
-0.33712	-3.42089
-0.19435	-1.64083
-0.10106	-0.29365
0.342684	-1.59953
-0.39038	-1.733
-0.03101	-2.18005
1.026377	-1.66134
1.662539	-0.39294
-0.16258	-0.86222
-0.50566	2.694623
0.558927	-2.90185
-0.34937	-1.55508
0.300248	-1.91379
-0.49764	-3.06643
-0.9177	-1.38577
0.679752	-2.68032
0.0579	-2.37497
0.224048	-2.32946
0.903519	-2.03945
0.14177	-1.53657
0.423911	-2.12047
0.279708	-0.95374
	-1.13034
	-2.7501
	-1.87473
	-2.07549
	-2.80836

0.41818	-3.09684
1.117091	-2.96403
0.586746	-3.86511
0.191949	-2.66139
0.907039	-3.77961
0.135494	-2.26763
0.3637	-2.57571
0.639899	-3.82235
0.76782	-3.8833
0.863154	-3.24957
0.656009	-2.02146
1.72664	-3.30414
0.435188	-3.44003
0.704954	-2.78155
0.214326	-3.857
0.470904	-4.07294
-0.3156	-3.93433
-0.23516	-3.96823
0.054483	-3.85349
0.114598	-4.08016
-0.13195	-2.81032
-0.19242	-2.89777
0.761042	-3.49353
0.410939	-2.68951
0.047304	-2.12137
0.12728	-1.69441
0.601093	-1.6722
-0.08811	-1.61467
0.075845	-2.18205
0.501994	-2.61902
0.438344	-2.15439
-0.01305	-2.48169
-0.32743	-1.93155
-0.2572	-2.21028
-0.34051	-2.35122
0.021679	-2.15423
-0.05829	-2.34831
-0.04229	-2.19491
-0.03018	-1.84463
0.014095	-2.31804
-0.35223	-1.92975
-0.53396	-1.83379
0.483809	-1.79066
-0.30713	-1.80483
0.702517	-1.50796
	-1.71959
	-2.15117
	-2.21717
	-1.96865
	-2.35584

-1.04103	-1.89979
-2.10804	-1.79095
-0.51409	-1.09436
-1.9966	-1.79052
-1.98323	-1.17236
-2.05081	-3.25361
-1.29687	-3.58027
-0.77731	-3.71431
-1.02799	-2.57132
-1.29559	-3.18881
-1.99851	-3.20229
1.36917	-3.36312
0.084018	-4.01351
-0.24204	-3.92742
0.791639	-3.97466
-1.41108	-3.38575
-1.13058	-3.12289
-1.62039	-3.41018
-1.13209	-2.18449
-2.41292	-2.16181
-0.98511	-1.6722
-2.00474	-1.57795
0.120316	-2.2961
-1.71051	-1.98759
-2.22295	-2.05333
-1.10168	-1.5877
3.272799	-2.92635
-1.3913	-3.03966
-1.5002	-3.81354
-2.10834	-3.62351
-2.22682	-3.46775
-2.58093	-3.65333
-1.14413	-3.66096
-2.71776	-3.02935
-1.69177	-3.21005
-1.55398	-3.34158
-1.41904	-2.46996
1.735813	-2.9172
3.600721	-4.08016
-2.72848	-3.37314
-1.29309	-3.38692
-2.01268	-2.90464
-0.90783	-2.51674
-2.8746	-2.94289
-1.65056	-2.57827
-1.04367	-3.70837
-0.18709	-1.53842
-3.19719	-1.19191
-0.65619	-1.29058
-2.88002	-1.21315

-1.45571	-1.22905
-1.42404	-0.46328
-0.76706	-0.99201
-1.82274	-0.41826
-1.54889	-1.70367
-1.08248	-0.8221
-1.85225	-0.53268
-2.64695	-0.47371
-3.219	-3.58624
-2.58208	-3.72364
-1.53471	-3.44408
-0.82373	-3.80928
-2.59685	-3.17232
-0.05887	-3.57011
-1.69645	-3.28683
-2.1255	-3.01217
-1.32721	-2.99196
-2.19864	-3.31421
-1.7865	-3.18705
-2.31923	-3.44456
-2.722	-3.51999
-2.76045	-4.02428
-2.68875	-3.27663
-2.64681	-3.2857
-1.92622	-2.59186
-2.31371	-2.86543
-2.01364	-3.47279

**Fig. 4b**	Low	Normal	Overexpression
Controls	2	95	3
Knockout	0	45	55

**Fig. 4c**	Low	Normal
Controls	2	91
Knockout	0	58

Overexpression	Fig. 4e	Controls	Knockout	Fig. 4g
7		-1.26228	1.660603	
42		-0.68916	0.65689	
		0.420267	0.24229	
		0.161956	-0.45101	
		0.795978	1.797767	
			1.065844	

Controls	Knockout
0	2.29
0	1.69
0	0.39
0	0.146
	0.57
	2.47

Fig. 4h

Controls	Knockout
0	14.166
0	9.327
0	3.339
	25.076
	0
	0

**Fig. 5b**	Controls	Knockout
	35.98828	31.25298
	36.90037	23.84957
	37.04126	28.72268
	33.81112	30.20159
	35.01331	

**Fig. 5d**	Controls	Knockout
	1142	1127
	1132	743
	907	729
	819	730
	1030.555	

Fig. 5e

Controls	Knockout	Fig. 5f	Controls	Knockout	Fig. 5g	Controls
985.114	1318.747		138.198	134.778		65.49155
1265.053	1075.759		145.62	101.764		100.7165
1316.225	1352.008		174.877	174.547		76.56419
869.398	1205.734		119.1	146.923		

Knockout
34.25635
46.12883
44.76151
73.99684

**Fig. 5h**	Controls	Knockout
	65	47
	48	34
	69	65
	47	42

**Fig. 5i**	Controls	Knockout
	9.473734	0
	25.31282	4.843764
	20.10145	0
	26.31406	11.38071

Fig. 6b	Controls	Knockout	Fig. 6d	Controls	Knockout	Fig. 6f
	55.08	99.76		79.55	138.41	
	57.6	95.17		69.54	246.23	
	45.9	71.49		86.6	198.16	
	77.7	87.25		98.92	155.62	
		62.26			236.46	

Controls	Knockout
29.25095	166.6774
11.57053	482.8927
84.64281	222.2442
13.53686	264.4642
13.563	

**Fig. 7a**	Controls	Knockout
	37.44601	39.0529
	34.0359	66.10588
	36.33032	47.48432
	28.13043	52.83299

**Fig. 7b**	Controls	Knockout
	26.97685	23.69337
	24.31812	20.55916
	28.30042	21.10167
	24.96135	17.89847

**Fig. 7c**	Controls	Knockout
In contact with Purkinje neurons	19	64
Not in contact	322	485

**Fig. 7d**	Controls
	150.9063
	133.296
	118.4052
	147.8471

Knockout	Fig. 7e	Controls	Knockout
266.3793		89.275	276.0313
216.9492		142.6	216.3521
224.4191		159.9788	285.2025
309.9477			

Fig. 8b

PV neurons	Non-PV cells
0.349069	-0.8803
1.331479	-0.52068
1.226866	-0.21094
-0.19338	-1.3386
-0.1172	-0.26325
0.559823	-1.62029
0.37936	-2.33557
-0.50286	0.506645
-1.15077	
-1.04835	
-1.35956	
0.977508	
1.098796	
-0.17185	
-0.24994	
0.084865	

Fig. 8c

PV neurons	Non-PV cells
0.176266	-1.49393
1.331287	-1.11306
2.344807	-0.54488
-0.78133	-1.04861
-0.94566	0.460732
0.239467	-1.70715
0.662784	-1.18361
-0.89898	-1.22725
-0.61936	
-1.04406	
-0.34657	
0.512999	
0.078713	
-0.19119	
0.217732	
0.404067	

**Fig. 8d**	PV neurons	Non-PV cells
	0.147235	-1.20266
	1.031837	-1.33579
	1.515062	-0.5659
	0.084912	-0.61973
	-0.85901	0.514977
	0.428816	-1.49015
	1.102418	-2.01327
	-0.82925	-0.6971
	-0.3736	
	-0.39381	
	-1.00723	
	-0.14178	
	-0.65212	
	0.283432	
	0.029104	
	-0.29788	

Fig. 9a

	Overexpression	Low	Deficient	Very Deficient
Controls	1	1	0	0
Patient 1 m.3243A>G	0	12	8	2
Patient 2 m.3243A>G	0	68	5	22
Patient 3 m.3243A>G	0	0	17	83
Patient 4 m.3243A>G	0	21	5	1
Patient 5 m.3243A>G	0	36	13	28
Patient 6 m.8344A>G	0	9	25	44
Patient 7 m.8344A>G	0	10	8	17
Patient 8 m.8344A>G	0	8	12	47
Patient 9 POLG	0	38	7	36
Patient 10 POLG	0	10	6	2
Patient 11 POLG	0	17	39	34

Fig. 9b

	Overexpression	Low	Deficient
Controls	2	1	0
Patient 1 m.3243A>G	0	0	0
Patient 2 m.3243A>G	0	32	5
Patient 3 m.3243A>G	0	17	83
Patient 4 m.3243A>G	0	5	3
Patient 5 m.3243A>G	0	5	0
Patient 6 m.8344A>G	0	16	15
Patient 7 m.8344A>G	0	10	8
Patient 8 m.8344A>G	0	4	25
Patient 9 POLG	0	19	2
Patient 10 POLG	0	2	0
Patient 11 POLG	0	27	14

Very Deficient
0
0
0
0
0
0
20
2
16
22
0
5

Fig. 9c

Controls	Mitochondrial disease
0.349069	-0.29164
1.331479	0.559553
1.226866	-0.49393
-0.19338	1.221222
-0.1172	1.383617
0.559823	-0.80329
0.37936	2.636893
-0.50286	1.03132
-1.15077	0.431601
-1.04835	2.193099
-1.35956	0.646441
0.977508	
1.098796	
-0.17185	
-0.24994	
0.084865	

Fig. 9e

Controls	Mitochondrial disease
42.36	22.37
40.34	25.55
43.03	11.93
35.53	28.87
28.76	36.16
33.4	18.46
37.67	32.44
33.88	
32.06	

Supp Fig. 1a	Controls	Knockout
	1178.08	629.91
	509.72	988.2
	1110.43	762.98
	385.64	1314.95
	1410.07	415.28
	991.49	556.07
	744.74	1211.78
	624.93	448.46
	765.62	431.67
	936.21	837.61
	547.2	367.46
	933.9	
	1170.94	
	811.76	
	241.27	
	2269.34	
	491.1	
	621.67	
	592.56	
	369.46	
	1574.58	
	187.33	
	513.99	
	575.75	
	536.93	
	663.55	
	625.23	
	393.25	

Supp Fig. 1b	Controls	Knockout
	1799.727	796.0373
	1034.677	496.8487
	1097.127	473.7321
	465.41	1294.318
	731.4871	253.2694
	356.6053	1377.119
	454.5609	648.0774
	410.234	482.2672
	366.1003	198.1559
	996.9908	251.6909
	249.9535	470.4613
	610.9007	
	651.6275	
	920.3051	
	320.4296	
	1238.753	
	438.3845	
	596.1032	
	313.7276	
	83.27403	
	1396.692	
	127.2306	
	433.4995	
	209.6987	
	464.2023	
	460.4167	

Supp Fig. 1c	Controls	Knockout
	790	651
	550	501
	638	517
	374	877
	555	607
	471	1200
	261	1591
	279	950
	460	1202
	785	
	270	
	633	
	946	
	537	
	207	
	994	
	670	
	506	
	365	
	769	
	923	
	230	
	381	
	248	

Supp Fig. 2

Controls	Knockout
2531611	313115.5
1437857	695939.6
4840959	593927.6
4489086	373871.6

Supp Fig. 3a	Controls	Knockout
	0.072961	-1.37697
	0.51903	0.279965
	-0.96112	-0.8122
	0.258461	2.070579
		0.038434
		0.367492

Supp Fig. 3b	Controls	Knockout
	-0.20886	-0.69844
	0.205247	-0.3741
	0.062693	0.964934
		0.827838
		1.62928

**Supp Fig. 3c**	Controls	Knockout
	-1.08846	-0.048
	-0.18961	0.000554
	0.713217	-0.04741
		-0.02446
		0.049225

**Supp Fig. 3d**	Controls	Knockout
	-0.27559	-0.6079
	0.414125	0.519403
	0.204826	0.411125
		0.645388
		1.855663

Supp Fig. 4a	Controls	Knockout	Controls	Knockout	Supp Fig. 4b	Controls	Knockout	Controls
	-0.189578	-4.31	0.032427	-3.22		-0.72395	-5.76	-0.16523
	-0.138345	-5.23	-0.191853	-4.32		0.368844	-4.63	-0.53163
	0.591799	-4.28	0.314412	-4.3		0.934484	-4.12	1.833859
		-4.08		-3.62			-5.4	
		-4.882967		-4.429943			-4.83307	
		-3.321285		-5.760242			-4.5914	

Knockout	Supp Fig. 4c	Controls	Knockout	Controls	Knockout	Supp Fig. 4d	Controls	Knockout
-1.95		0.603818	-2.644166	0.407027	0.791907		1.289752	-1.0118
-2.11		-0.38553	-4.169305	-0.0765	-1.91271		-0.69582	-0.77554
-1.73		-0.39812	-3.800755	-0.47945	-1.885415		0.483765	-2.24858
-2.53		0.772832	-3.208331	1.2526	-1.147437			-2.6622
-4.14194			-3.11224		-1.42095			-1.4683
-1.07925			-2.07428		-2.69181			

Controls	Knockout	**Supp Fig. 4e**	Controls	Knockout	Controls	Knockout	**Supp Fig. 4f**	Controls
-0.02239	-1.08217		1.461998	0.117134	0.644879	2.2538		-1.00874
-0.06669	-1.42626		-0.4567	-2.58315	-0.58525	-0.26535		0.512546
0.452395	-0.79786		-0.63348	-2.64932	-0.13809	-1.27163		-0.4353
-0.35461	-0.62032			-3.89185		-0.8773		
1.912801	-1.07709			-2.23635		0.646472		
	-2.77788							

Knockout	Controls	Knockout
0	0.20297	1.06
-1.02	-0.13985	-0.65
-0.37	0.154903	-0.01
0.57		0.46
0.143933		-2.88957
-2.33583		0.456453

Supp Fig. 5a	Controls	Knockout
	0.090726	0.237271
	0.162095	0.136786
	-0.468142	-0.040418
		-1.307514
		-0.579229
		0.190794

Supp Fig. 5b	Controls	Knockout
	-0.26429	0.411228
	0.149609	-0.28024
	0.301781	-2.05673
		1.317067
		-0.58834
		0.214141

0.162095

-0.468142

**Supp Fig. 5c**	Controls	Knockout
	-0.0218	0.428761
	-0.35333	0.21512
	0.484389	-0.56241
		-0.21564
		0.920243

**Supp Fig. 5d**	Controls	Knockout
	0.854468	0.568754
	1.247484	0.483366
	-0.61709	-0.14435
	-0.4311	0.731001
	1.112757	0.094293
		-1.53014

**Supp Fig. 5e**	Controls	Knockout
	-0.15331	0.184683
	0.576594	0.047191
	-0.30009	-0.16641
	-0.25574	-1.8998
		-0.72183
		0.204242

**Supp Fig. 5f**	Controls
	-0.38912
	0.259892
	-0.28191

Knockout
1.227126
-0.56198
-2.42838
1.644843
-0.14908
0.104695

Supp Fig. 7b

Controls	Knockout
-0.22713	0.434912
-0.56119	0.553395
-0.39783	0.527177
-0.2276	0.466434
-0.49055	0.279291
-0.25834	0.329914
-0.80217	0.135384
-0.77838	-0.3612
-1.36112	-0.13555
-0.72167	0.036479
-0.75061	-0.01702
-0.83744	0.220876
-1.78228	0.194819
-1.16127	-0.37572
-1.62649	0.200049
-1.28127	0.211463
-1.22705	0.395972
-1.27176	0.013127
-1.13683	-0.11816
-1.47101	0.014022
-1.5195	0.025638
-1.16933	0.049907
-1.5606	-0.13586
-1.31224	0.122422
-1.34548	-0.07401
-1.38685	0.098104
-1.15341	0.16448
-1.12429	0.018029
-1.79714	-0.47962
-1.52232	-0.14151
-1.50945	-0.84792
-1.59994	-0.12905
-1.85616	-0.54265
0.825116	-0.33603
1.285578	-0.58659
1.25935	-0.23449
1.230892	0.071364
1.414714	-0.10407
1.893514	-0.37247
1.91669	-0.37663
1.718895	-0.77043
1.056971	-0.48388
0.782656	-0.41161
0.673232	-0.39951
0.296694	-0.58442
0.137555	-0.82177
-0.00122	-0.50991
0.117138	-0.58026
-0.08281	-0.58723

Supp Fig. 7c

Controls	Knockout
-0.23332	0.449397
-0.61571	0.506083
-0.10241	0.234137
-0.44334	0.061739
-0.20311	0.539447
-0.62846	-0.21278
-1.08112	0.505942
-0.90547	-0.00501
-0.91683	0.085787
-1.02886	0.414462
-1.14064	0.010257
-0.84063	0.059799
-1.36658	0.206252
-1.59899	-0.52283
-2.11287	0.716711
-0.94753	0.211141
-1.27416	0.179254
-1.52016	-0.50684
-1.15439	-0.1149
-1.68746	0.002069
-1.46238	0.118419
-1.30589	0.301987
-1.80788	-0.144
-1.35054	0.444534
-1.27518	-0.04795
-0.9811	0.633632
-1.43687	0.449157
-1.20803	0.370062
-1.33063	-0.06856
-1.39477	0.159644
-1.6265	-0.14229
-1.58973	0.23588
-1.66787	-0.4693
0.88324	0.490336
1.318568	-1.0245
0.810416	-0.40309
1.169405	0.335507
0.973246	0.227261
1.413931	-0.67903
1.452539	-0.25408
1.887462	0.046331
0.844578	-0.11725
0.233894	-0.34962
0.523294	-0.40993
0.027229	0.107276
0.059365	0.14754
0.300109	-0.0313
0.006064	-0.12659
-0.08644	-0.86469

0.035676	-0.35884	0.339409	0.256229
0.931278	-0.72748	0.724429	-0.6318
1.644933	-0.48438	1.232444	-0.0239
1.262579	-0.31351	1.069869	-0.11858
0.511035	-0.35671	0.518348	0.264374
-0.36131	-0.31951	-0.3183	0.1806
0.066363	0.036007	0.325851	-0.35894
-0.70585	-0.55117	-0.92859	-0.50547
0.062659	0.27743	0.127639	1.190528
0.169885	0.444296	-0.07517	0.724481
-0.59216	0.041953	-0.33231	0.435158
-0.36332	0.242074	-0.56802	-0.86066
-0.64352	-0.0762	-0.59293	-0.133
-0.26878	-0.1962	-0.60221	-0.3062
-0.3158	0.190021	-0.62878	0.473455
-1.17495	0.502481	-1.14096	0.653412
-0.46638	-0.0026	-0.23092	0.309465
-0.6271	0.077684	-0.18715	0.367926
-0.90307	0.248685	-0.9609	0.037727
-0.79517	0.680619	-0.86785	1.008106
-0.80387	0.467927	-0.16933	0.793311
-0.60246	0.450133	0.059174	0.619857
0.135667	0.25255	0.012473	0.190783
0.182462	-0.07856	-0.15506	-0.64603
-0.1927	-0.3913	-0.00784	-0.15064
-0.06102	-0.0677	0.093389	0.145029
-0.04882	-0.11229	-0.00331	-0.36659
-0.08023	-0.06588	-0.16284	-0.42618
0.096896	-0.81216	0.759189	-1.17356
0.441293	-1.22681	0.192801	-1.78352
-0.44334	-0.89642	-0.29808	-0.23012
-0.52605	-0.71863	-0.25098	-0.35946
-0.70558	-0.25427	0.34142	0.121354
-0.64958	-0.27109	-0.36206	-0.04768
-0.82203	-1.38817	-0.60954	-0.71271
0.565839	0.853954	0.810285	0.972823
0.167957	1.430711	-0.66427	1.00222
0.246871	1.77664	0.099828	1.690395
-0.10217	1.520443	0.580534	1.613138
0.134721	1.336469	-0.02169	2.031426
0.082763	1.069409	-0.37054	1.527834
0.421615	1.016176	-0.08899	1.832719
0.83533	1.289028	0.28862	1.730529
0.798741	1.781051	1.017514	1.941255
0.286667	1.645831	0.707663	1.47511
0.26562	1.821924	0.14193	1.992639
0.674839	-0.35964	1.337062	-0.22994
1.440999	0.019739	1.236333	0.094753
1.46304	0.309051	1.595429	-0.07101
1.235844	0.218847	1.077317	0.482996

1.023531	0.045129		0.932327	0.118978
1.513145	0.442927		1.631926	0.261254
1.687766	0.076897		1.66505	-0.16655
1.20648	-0.13247		1.043476	0.23241
1.286381	0.347375		1.584878	0.534366
0.894686	0.284019		0.920902	-0.09455
1.768413	0.541687		1.702285	0.807078
1.40471	0.28408		1.176297	0.654511
1.015818	-0.04178		1.379559	-0.04701
1.487771	-0.25833		1.075174	-0.29185
1.005813	0.538818		1.295063	0.393537
0.140532	0.990879		1.663987	0.764934
0.932157	0.800471		0.516524	0.857823
1.190737	0.634896		1.278988	0.729342
0.763262	0.674852		1.647769	0.50079
1.123884			1.30006	
0.943364			0.823175	
0.925661			0.75548	
0.483337			-0.06074	

Supp Fig. 7d

Controls	Knockout
-1.627696	0.294132
-1.269792	0.615143
-1.380824	0.640664
-1.606247	0.468145
-1.649491	0.578098
-1.40197	0.590728
-1.374039	0.43465
-1.083251	0.431415
-1.544909	0.458993
-1.332295	0.276599
-1.578075	0.484407
-1.277631	0.426826
-1.165196	0.296688
-1.256108	0.676094
-1.368025	0.858789
-1.249015	0.548218
-1.713757	0.764214
-1.742143	0.4586
-1.648683	0.502775
-1.598785	1.163263
-1.670436	0.844153
-1.917917	0.738291
-0.846035	0.48203
-0.498348	0.302582
-0.854162	0.427709
-0.646303	0.559369
-0.318202	0.930132
-0.887861	1.256723
-1.080376	1.112295
-0.88972	0.974171
-1.026071	1.096565
-0.902713	0.88705
-0.957263	0.92813
-0.598813	1.095428
-0.580273	1.00479
-0.81202	0.751352
-0.376377	0.628897
-0.648823	0.414832
-0.76626	1.322988
-0.786355	0.877244
-1.147264	1.502452
-0.804626	0.953402
-0.958743	1.382518
-0.138843	1.307205
-0.707914	1.357048
-0.566196	0.668244
0.004397	0.878627
-0.394586	1.258279
-0.804523	1.007909

Supp Fig. 7e

Controls	Knockout
-1.68829	-0.23156
-0.76581	0.134855
-0.78586	0.343121
-1.38183	0.701404
-1.5153	0.612008
-1.33632	0.182495
-1.14266	-0.05544
-0.52155	0.015063
-1.19102	0.870958
-0.83138	-0.21018
-1.40721	0.644888
-1.28741	-0.0717
-0.60064	0.500947
-1.02239	1.121986
-0.43803	1.269028
-0.9729	1.094983
-1.7426	0.558072
-1.84673	1.192526
-1.71265	1.029445
-1.68494	1.025159
-1.95778	0.330619
-1.81301	0.931817
-1.0239	0.594774
-1.01628	0.883524
-1.42951	0.555689
-2.24442	0.703639
-0.99468	0.487042
-1.55655	1.728396
-2.00271	1.758682
-1.77856	0.826766
-1.23054	1.091149
-1.53722	0.830461
-2.01942	0.546461
-0.68676	0.774186
-0.9016	0.78543
-0.7526	0.425921
0.121047	0.295321
-0.87045	-0.34717
-1.135	1.585878
-0.52697	0.328625
-1.69339	1.694677
-0.44203	0.394413
-1.22006	1.393748
-0.37635	1.080476
-1.1371	1.954188
-0.02992	0.573839
0.411937	0.604608
0.412156	1.111271
-0.7079	0.834015

0.1127	1.113189	-0.19248	1.308143
-0.512009	1.131025	-0.95162	1.639054
-0.46835	0.991928	0.101067	1.078405
-0.60179	0.942021	-0.75801	0.836556
-0.903968	1.268616	-0.3769	1.984358
-0.447138	1.123226	0.331847	0.971444
0.266091	0.984291	0.919843	0.774534
-0.132376	1.316902	1.174324	1.388421
0.021248	1.53274	0.876709	2.35872
-0.088806	1.73192	0.323055	2.275348
0.064598	1.02263	0.534247	1.270093
0.00365	1.724354	0.093304	2.492202
0.195778	1.686942	1.04904	2.014569
0.313088	1.338555	1.209924	1.555217
0.246205	1.13072	0.703237	1.456964
0.376622	1.051029	1.129249	0.84706
-0.036327	1.180443	0.204888	1.838667
-0.079792	1.338098	0.712682	2.159232
-0.433418	1.06512	-0.29429	1.023944
-0.207658	1.271864	-0.19604	1.261784
-0.225606	1.683969	-0.26433	2.506397
0.101842	1.488456	1.306808	1.702508
-0.082061	1.904783	0.284243	2.844197
0.066838	1.46966	0.452839	2.237652
-0.087496	1.36996	0.317535	1.610586
-0.292965	1.529648	0.231871	2.089607
-0.589594	1.349283	-0.70516	2.805049
0.210314	1.65895	1.088181	2.241405
0.025909	1.602417	0.145161	1.720711
0.189027	1.627351	0.25522	2.049754
0.030019	1.265834	0.025335	0.611811
0.28924	1.503759	0.424876	1.675802
-0.130216	1.344984	0.544082	2.343046
-0.216854	1.385303	0.013449	1.323864
0.28132	1.809995	0.612331	2.022566
-0.348711	1.618967	-0.28072	1.829446
0.105205	1.176342	0.583545	1.396033
-0.063423	1.248023	1.03845	1.236518
0.268855	1.279202	1.241228	2.019173
0.312747	1.836112	1.380819	2.274354
-0.595442	1.680705	-0.8732	2.070414
-0.315574	1.160396	-0.16284	1.179173
-0.223816	1.604766	0.032354	1.535868
-0.156628	1.43422	0.193759	2.131492
0.005031	1.620143	0.509991	2.099348
0.082357	1.479636	0.467479	1.804148
0.183484	1.602455	0.952496	1.414659
-0.187698	1.396903	0.21896	1.414063
0.364619	1.481522	1.393272	1.856253
0.407444	1.506548	0.990686	1.376204

0.207079	1.460641	0.568029	2.394681
0.426347	1.476435	1.490294	2.031855
0.1433	1.277318	0.619996	1.446164
0.261911	0.878693	1.309379	1.035683
0.170898	1.194551	0.367389	1.231026
0.072384	1.718402	0.704814	1.90341
0.241285	1.260771	0.8948	1.113045
0.699055	2.802388	0.225296	2.621384
1.397985	1.761176	1.261187	2.310689
1.667968	1.847971	1.528937	2.191201
1.64878	1.464627	1.266366	1.589704
1.461482	1.769601	1.655311	2.364717
1.30966	1.474333	1.041415	1.536202
1.528661	1.760692	1.306016	2.302603
1.473222	1.548606	1.216186	1.430521
1.534945	1.377107	1.29968	0.72915
1.617333	1.51794	1.382228	1.99638
1.535111	1.480907	0.804729	1.001901
1.666485	1.555651	1.514715	1.392431
1.811622	1.162431	1.718727	0.732754
1.309058	1.61478	1.303982	1.805837
1.78301	1.518557	1.170836	1.143883
1.46579	1.651005	0.340195	2.180792
1.52972	1.576852	1.286888	1.87454
1.02453	1.813314	0.605068	1.949543
1.24795	1.876715	0.2251	2.417072
1.332223	2.139023	0.748608	2.353996
1.416969	2.398917	0.593503	2.759802
1.411773	1.296011	1.2614	0.94996
1.192344	1.534896	-0.02868	1.569913
1.193717	1.261119	0.18479	1.461905
1.058553	1.451772	1.056616	1.015407
1.773367		1.043689	
1.837035		1.002029	
1.401234		0.176416	
0.919425		0.297235	
1.056715		-0.22211	
0.960902		0.01776	
1.3035		0.288993	
1.264107		0.02196	
0.931261		0.14802	
1.065623		-0.00206	
1.269871		0.039911	
0.732368		-0.21717	
1.606341		0.367033	
1.391136		1.116815	

Supp Fig. 8a	Controls	Knockout
	0.517326	0.175487
	-0.11757	1.732203
		-2.09345

Supp Fig. 8b	Controls	Knockout
	-0.94574	0.640683
	0.236434	0.113919
		-1.15862
		2.379752

Supp. Fig. 9

Controls	Patients
-0.01451	-0.69231
-0.06024	-1.23169
-0.0827	-0.49706
0.288763	-0.95708
-1.44205	-0.24797
-0.35742	-1.21308
0.119372	0.925381
-1.14707	0.059885
-0.16315	-1.09204
-0.23596	-1.10517
0.61494	-0.7368
0.768312	
1.300418	
-1.55783	
0.375888	
0.778998	
