# Supplementary material for: A novel mouse model of mitochondrial disease exhibits juvenile-onset severe neurological impairment due to parvalbumin cell mitochondrial dysfunction
Source: Commun Biol. 2023 Oct 23;6:1078. doi: 10.1038/s42003-023-05238-7 (PMC10593770; doi:10.1038/s42003-023-05238-7)
Supplement: Supplementary file 5 — Reporting Summary [file 42003_2023_5238_MOESM5_ESM.pdf]

nature portfolio

Corresponding author(s): Grainne Gorman COMMSBIO-22-3450-T

Last updated by author(s): Jun 7, 2023

Reporting Summary

Nature Portfolio wishes to improve the reproducibility of the work that we publish. This form provides structure for consistency and transparency in reporting. For further information on Nature Portfolio policies, see our Editorial Policies and the Editorial Policy Checklist.

Statistics

For all statistical analyses, confirm that the following items are present in the figure legend, table legend, main text, or Methods section.

n/a	Confirmed	
☐	☒	The exact sample size (*n*) for each experimental group/condition, given as a discrete number and unit of measurement
☐	☒	A statement on whether measurements were taken from distinct samples or whether the same sample was measured repeatedly
☐	☒	The statistical test(s) used AND whether they are one- or two-sided<br>*Only common tests should be described solely by name; describe more complex techniques in the Methods section.*
☒	☐	A description of all covariates tested
☐	☒	A description of any assumptions or corrections, such as tests of normality and adjustment for multiple comparisons
☐	☒	A full description of the statistical parameters including central tendency (e.g. means) or other basic estimates (e.g. regression coefficient) AND variation (e.g. standard deviation) or associated estimates of uncertainty (e.g. confidence intervals)
☐	☒	For null hypothesis testing, the test statistic (e.g. *F*, *t*, *r*) with confidence intervals, effect sizes, degrees of freedom and *P* value noted<br>*Give P values as exact values whenever suitable.*
☒	☐	For Bayesian analysis, information on the choice of priors and Markov chain Monte Carlo settings
☒	☐	For hierarchical and complex designs, identification of the appropriate level for tests and full reporting of outcomes
☒	☐	Estimates of effect sizes (e.g. Cohen's *d*, Pearson's *r*), indicating how they were calculated

Our web collection on statistics for biologists contains articles on many of the points above.

Software and code

Policy information about availability of computer code

Data collection	MBF Biosciences StereoInvestigator v.9<br>Nikon NIS Elements<br>Carl Zeiss Zen (Black Edition) v.2.3 SP1<br>CFX96 Touch Real-Time PCR Detection System (Bio-rad)<br>ImageLab software (Bio-Rad) v.4.1<br>Carl Zeiss PALM RoboSoftware v.4.6
Data analysis	Fiji (ImageJ) v.1.51-1.53<br>Zeiss Zen (Blue, Lite Edition) v.2, v.3.2, v.3.3<br>MBF Biosciences StereoInvestigator v.9<br>CFX Manager (Bio-rad) v.3.1<br>GraphPad Prism v.8-v.9.5.1<br>R 4.2.2<br>RStudio 2022.12.0<br>Volocity (Perkin Elmer) v.6.1<br>ImageLab software (Bio-Rad) v.6.0<br>Microsoft Excel 2018-2022

For manuscripts utilizing custom algorithms or software that are central to the research but not yet described in published literature, software must be made available to editors and reviewers. We strongly encourage code deposition in a community repository (e.g. GitHub). See the Nature Portfolio guidelines for submitting code & software for further information.

Data

Policy information about availability of data

All manuscripts must include a data availability statement. This statement should provide the following information, where applicable:

- Accession codes, unique identifiers, or web links for publicly available datasets
- A description of any restrictions on data availability
- For clinical datasets or third party data, please ensure that the statement adheres to our policy

The data that support the findings of this study are available from the corresponding author upon reasonable request.

Human research participants

Policy information about studies involving human research participants and Sex and Gender in Research.

Reporting on sex and gender	We report biological sex in the study of the patients and controls. Two Female and nine Male patients were within the cohort. Due to a low sample size in the female group and heterogeneity of genetic diagnoses, we were not able to perform any statistical testing between the sexes.
Population characteristics	Eleven human patients with a genetically and clinically confirmed diagnosis of mitochondrial disease were included in the study. Five cases harbored m.3243A>G, three m.8344A>G, three recessive biallelic POLG pathogenic variants. Age range 30-79. 2 Female, 9 Male. Age and sex was matched for the control cohort included in this study.
Recruitment	Informed consent was provided prior to brain organ donation post-mortem. Samples were coded to maintain confidentiality. We do not anticipate any self-reflection bias, as the diagnosis was confirmed genetically and clinically antemortem.
Ethics oversight	Tissues were obtained from the Newcastle Brain Tissue Resource (NBTR) with ethical approval from the Joint Ethics Committee of Newcastle and North Tyneside 1 REC (Ref 19/NE/0008) as well as Edinburgh Brain bank which has ethical approval from the East of Scotland Research Ethics Service REC1.

Note that full information on the approval of the study protocol must also be provided in the manuscript.

Field-specific reporting

Please select the one below that is the best fit for your research. If you are not sure, read the appropriate sections before making your selection.

☒ Life sciences ☐ Behavioural & social sciences ☐ Ecological, evolutionary & environmental sciences

For a reference copy of the document with all sections, see nature.com/documents/nr-reporting-summary-flat.pdf

Life sciences study design

All studies must disclose on these points even when the disclosure is negative.

Sample size	No statistical methods were used to predetermine sample size. Sample sizes were chosen based on previous studies which had shown robust statistical power.
Data exclusions	No data were excluded.
Replication	For all experiments, a minimum of n = 3-4 mice per group were used to ensure a sufficient number of biological replicates. For all staining procedures, a sufficient number of neurons was analyzed to control for biological variability between cases. Image analysis was performed at the same time for each experiment. Due to the high volume of optimization and staining experiments, these were not repeated due to scarcity of post-mortem brain tissues and to minimize the number of animals used in research.
Randomization	Mice were allocated to the experimental groups based on their genotype.
Blinding	Investigators were blinded to the genotype upon carrying out behavioral tests and analyzing behavioral data, however, due to the severity of the motor phenotype in the mouse model, in some instances blinding was not possible. Blinding was not done for staining experiments analysis as the neurons were randomly selected based on the cell marker within the brain region of interest and protein expression was analyzed following image capture which was consistent for all images.

Reporting for specific materials, systems and methods

We require information from authors about some types of materials, experimental systems and methods used in many studies. Here, indicate whether each material, system or method listed is relevant to your study. If you are not sure if a list item applies to your research, read the appropriate section before selecting a response.

Materials & experimental systems

n/a	Involved in the study
☐	☒ Antibodies
☒	☐ Eukaryotic cell lines
☒	☐ Palaeontology and archaeology
☐	☒ Animals and other organisms
☒	☐ Clinical data
☒	☐ Dual use research of concern

Methods

n/a	Involved in the study
☒	☐ ChIP-seq
☒	☐ Flow cytometry
☒	☐ MRI-based neuroimaging

Antibodies

Antibodies used

mouse anti-NDUFB8 antibody [clone 20E9DH10C12] (ab110242, Abcam), 1:100 (IF)
mouse anti-GRIM19 antibody [clone 6E1BH7] (ab110240, Abcam), 1:100 (IF)
mouse anti-UQCRC2 antibody [clone 13G12AF12BB11] (ab14745, Abcam), 1:100 (IF)
mouse anti-SDHA antibody [clone 2E3GC12FB2AE2] (ab14715, Abcam), 1:200 (IF)
mouse anti-MTCO1 antibody [clone 1D6E1A8] (ab14705, Abcam), 1:200 (IF)
mouse anti-COXIV antibody [clone 20E8C12] (ab14744, Abcam), 1:200 (IF)
mouse anti-VDAC1/Porin antibody [clone 20B12AF2] (ab14734, Abcam), 1:200 (IF)
mouse anti-parvalbumin antibody [clone PARV19] (P3088, Sigma Aldrich), 1:100 (IF), 1:2000 (human IHC)
mouse anti-calbindin (300, Swant), 1:6000 (IHC)
rabbit anti-c-Fos (ab222699, Abcam), 1:2000 (IHC)
rabbit anti-pyruvate carboxylase antibody (HPA043922, Sigma Aldrich), 1:100 (IF)
rabbit anti-PGC1 antibody (AB3242, Sigma Aldrich), 1:100 (IF)
rabbit anti-parvalbumin antibody (PV27, Swant), 1:100 (mouse IF), 1:500 (mouse IF, if biotinylated), 1:1500 (human IF), 1:6000 (mouse IHC)
rabbit anti-Iba1 antibody (019-19741, Alpha Laboratories Ltd), 1:2000 (IHC), 1:100 (IF)
rabbit anti-GFAP antibody (Z0334, Dako), 1:15000 (IHC)
rabbit anti-GAD65-67 antibody (G5163, Sigma-Aldrich), 1:100 (IF)
rabbit anti-tyrosine hydroxylase antibody (T8700, Sigma-Aldrich), 1:100 (IF)
goat anti-rabbit IgG (H+L) biotinylated antibody (BA1000, Vector), 1:200
goat anti-mouse IgG (H+L) biotinylated antibody (BA9200, Vector), 1:200
streptavidin, Alexa Fluor 405 conjugated antibody (S32315, Invitrogen), 1:200
goat anti-rabbit IgG (H+L) Alexa FLuor 350 (A11046, Invitrogen), 1:100
goat anti-rabbit IgG (H+L) Alexa Fluor 405 conjugated antibody (A11008, Invitrogen), 1:100
goat anti-rabbit IgG (H+L) Alexa Fluor 546 conjugated antibody (A11010, Invitrogen), 1:100
goat anti-mouse IgG1 Alexa Fluor 647 conjugated antibody (A21240, Invitrogen), 1:100
goat anti-mouse IgG2b Alexa Fluor 647 conjugated antibody (A21242, Invitrogen), 1:100
goat anti-mouse IgG2b Alexa Fluor 488 conjugated antibody (A21141, Invitrogen), 1:100
goat anti-mouse IgG2a Alexa Fluor 546 conjugated antibody (A21131, Invitrogen), 1:100
goat anti-mouse IgG2a Alexa Fluor 488 conjugated antibody (A21131, Invitrogen), 1:100
goat anti-mouse IgG2b Alexa Fluor 546 conjugated antibody (A21143, Invitrogen), 1:100
goat anti-rabbit IgG (H+L) Alexa Fluor 405 conjugated antibody (A48254, Invitrogen), 1:100

Validation

All primary antibodies used were commercially available and validated by the manufacturers and have been extensively used in previous publications. Links to manufacturers' websites that contain relevant references are below. No-primary-antibody controls (secondary antibody-only) were used to check for any non-specific labeling in optimisation stages and/or experiments. Cellular localization of the antibody labeling was confirmed to be as predicted, e.g., anti-pyruvate carboxylase and anti-OXPHOS antibodies showed cytoplasmic staining and anti-c-Fos antibody demonstrated a nuclear staining.

mouse anti-NDUFB8 antibody (ab110242, Abcam): https://www.abcam.com/ndufb8-antibody-20e9dh10c12-ab110242.html
mouse anti-GRIM19 antibody (ab110240, Abcam): https://www.abcam.com/grim19-antibody-6e1bh7-ab110240.html
mouse anti-UQCRC2 antibody (ab14745, Abcam): https://www.abcam.com/uqcrc2-antibody-13g12af12bb11-ab14745.html
mouse anti-SDHA antibody (ab14715, Abcam): https://www.abcam.com/sdha-antibody-2e3gc12fb2ae2-ab14715.html
mouse anti-MTCO1 antibody (ab14705, Abcam): https://www.abcam.com/mtco1-antibody-1d6e1a8-ab14705.html
mouse anti-COXIV antibody (ab14744, Abcam): https://www.abcam.com/cox-iv-antibody-20e8c12-ab14744.html
mouse anti-VDAC1/Porin antibody (ab14734, Abcam): https://www.abcam.com/products/primary-antibodies/vdac1porin-antibody-20b12af2-ab14734.html
mouse anti-parvalbumin antibody (P3088, Sigma Aldrich): https://www.sigmaaldrich.com/GB/en/product/sigma/p3088
mouse anti-calbindin (300, Swant):
rabbit anti-c-Fos (ab222699, Abcam): https://www.abcam.com/nav/primary-antibodies/rabbit-monoclonal-antibodies/c-fos-antibody-epr21930-238-ab222699.html
rabbit anti-pyruvate carboxylase antibody (HPA043922, Sigma Aldrich): https://www.atlasantibodies.com/products/antibodies/primary-antibodies/triple-a-polyclonals/pc-antibody-hpa043922/
rabbit anti-PGC1 antibody (AB3242, Sigma Aldrich): https://www.merckmillipore.com/GB/en/product/Anti-PGC-1-Antibody,MM_NF-AB3242
rabbit anti-parvalbumin antibody (PV27, Swant): https://www.swant.com/pdfs/PV27_Rabbit_anti_Parvalbumin.pdf
rabbit anti-Iba1 antibody (019-19741, Alpha Laboratories Ltd): https://labchem-wako.fujifilm.com/us/product/detail/

W01W0101-1974.html
rabbit anti-GFAP antibody (Z0334, Dako): https://www.agilent.com/en/product/immunohistochemistry/antibodies-controls/primary-antibodies/glial-fibrillary-acidic-protein-%28concentrate%29-76683
rabbit anti-GAD65-67 antibody (G5163, Sigma-Aldrich): https://www.sigmaaldrich.com/GB/en/product/sigma/g5163
rabbit anti-tyrosine hydroxylase antibody (T8700, Sigma-Aldrich): https://www.sigmaaldrich.com/GB/en/search/t8700?focus=products&page=1&perpage=30&sort=relevance&term=t8700&type=product

Animals and other research organisms

Policy information about studies involving animals; ARRIVE guidelines recommended for reporting animal research, and Sex and Gender in Research

Laboratory animals	Transgenic strains were all on a C57BL/6 background (B6;129P2 Pvalbtm1(cre)Arbr and C57/BL/6J TFAMloxP). Mice used in the study were typically between 5 weeks and 13 weeks of age. Both female and male mice were used in the study. Mice were housed in single sex cages. Mice were not single-housed in this study. Mice were placed on the normal 12-h light/dark cycle with lights on at 07:00 with food and water provided ad libitum.
Wild animals	The study did not involve wild animals.
Reporting on sex	Both sexes were used in the study. The sex of animals was assessed by technicians. Data was not segregated according to sex of the animals, as there were no differences in phenotype presentation between male or female mice. Both sexes were pooled together to one experimental group based on the genotype.
Field-collected samples	The study did not involve animals collected from the field.
Ethics oversight	All animal experiments were conducted in compliance with the UK Home Office and Newcastle University Centre for Comparative Biology (CBC) Animal Welfare Ethical Review Board (AWERB).

Note that full information on the approval of the study protocol must also be provided in the manuscript.
